# hnRNPU Safeguards Oocyte Development and Female Fertility via Regulation of Alternative Splicing

**DOI:** 10.1096/fj.202503270R

**Published:** 2026-01-12

**Authors:** Jinmei Li, Bei Chen, Yujiao Wen, Yanqing Wu, Kuan Liu, Jingshou Chen, Shuangqi Wang, Shenglei Feng, Juan Dong

**Affiliations:** ^1^ Department of Obstetrics and Gynecology Union Hospital, Tongji Medical College, Huazhong University of Science and Technology Wuhan China; ^2^ Department of Obstetrics and Gynecology Xiangya Hospital, Central South University Changsha China; ^3^ Reproductive Medical Center Renmin Hospital of Wuhan University Wuhan Hubei China; ^4^ Department of Gynecology Maternal and Child Health Hospital of Hubei Province, Tongji Medical College, Huazhong University of Science and Technology Wuhan China; ^5^ Institute of Reproductive Health, Tongji Medical College, Huazhong University of Science and Technology Wuhan China; ^6^ Department of Comparative Biosciences University of Illinois at Urbana‐Champaign Urbana Illinois USA; ^7^ Department of Biomedical Sciences College of Biology, Hunan University Changsha China

**Keywords:** alternative splicing, female, heterogeneous nuclear ribonucleoprotein U, infertility, mitochondria, oocyte

## Abstract

RNA‐binding proteins (RBPs) are essential for oocyte development, folliculogenesis, and ovarian homeostasis by regulating RNA metabolism. Heterogeneous nuclear ribonucleoprotein U (hnRNPU) plays critical roles in the regulation of multiple physiological processes as a key RBP, yet its function in mammalian oocytes remains poorly understood. Here, we generated two oocyte‐specific *Hnrnpu* knockout mouse models by using *Zp3*‐Cre and *Gdf9*‐Cre transgenic mouse lines to dissect its role in female reproduction. Both models exhibited severe follicular developmental arrest and complete female infertility. *Zp3*‐Cre‐mediated deletion caused oocyte arrest at the germinal vesicle (GV) stage, whereas *Gdf9*‐Cre‐mediated deletion nearly abolished GV oocyte retrieval, indicating a more severe developmental block and revealing stage‐specific requirements for hnRNPU. Mechanistic investigations focused on the *Zp3*‐Cre model, which provided sufficient GV oocytes for molecular analyses. Loss of *Hnrnpu* in growing oocytes led to mitochondrial dysfunction, impaired oocyte‐granulosa cell communication, and increased follicular apoptosis. Transcriptomic profiling revealed widespread dysregulation of genes involved in mitochondrial function and cell adhesion, with numerous mitochondrial‐associated transcripts exhibiting aberrant pre‐mRNA splicing. Collectively, our findings identify hnRNPU as an indispensable regulator of oocyte development and female fertility, acting through alternative splicing regulation to preserve mitochondrial function, maintain oocyte quality, and support folliculogenesis.

## Graphical abstract

Heterogeneous nuclear ribonucleoprotein U (hnRNPU) is essential for oocyte development and female fertility. Oocyte‐specific deletion of *Hnrnpu* leads to follicular arrest, impaired meiotic maturation, mitochondrial dysfunction, and infertility. Mechanistically, hnRNPU preserves oocyte quality and supports folliculogenesis by regulating the transcription of mitochondrial and oocyte‐granulosa cell communication genes, as well as controlling the splicing of key mitochondrial transcripts.

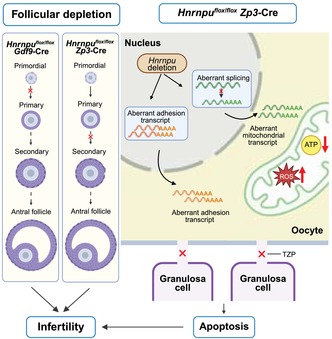

**Abbreviations**
A3SS: alternative 3′ splice sites
A5SS: alternative 5′ splice sites
AS: alternative splicing
cAMP: cyclic adenosine monophosphate
cGMP: cyclic guanosine monophosphate
DEGs: differentially expressed genes
FSH: follicle‐stimulating hormone
GCs: granulosa cells
GO: gene ontology
GV: germinal vesicle
GVBD: germinal vesicles nuclear membrane breakdown
hCG: human chorionic gonadotrophin
hnRNP: heterogeneous nuclear ribonucleoprotein
MI: metaphase I
MII: metaphase II
MXE: mutually exclusive exons
OMM: outer mitochondrial membrane
PMSG: pregnant mare serum gonadotropin
POI: premature ovarian insufficiency
RBPs: RNA‐binding proteins
RI: retained introns
ROS: reactive oxygen species
SE: skipped exon
TZPs: transzonal projections

## 1 Introduction

In mammals, female reproductive longevity is limited by the finite ovarian follicle pool [1]. Oocytes originate from primordial germ cells and develop within follicles, where they undergo a series of tightly regulated developmental events. In mammalian post‐natal ovaries, oocytes arrest at the diplotene stage of prophase I, known as the germinal vesicle (GV) stage. During this period, they acquire meiotic competence by synthesizing and storing maternal RNAs and proteins essential for subsequent maturation [2]. Following a luteinizing hormone (LH) surge, GV‐stage oocytes resume meiosis, initiating chromatin condensation and nuclear envelope breakdown. This transition marks entry into meiosis I (MI), characterized by spindle assembly and chromosome alignment. Completion of MI is signaled by extrusion of the first polar body, after which oocytes proceed to meiosis II (MII) and arrest at metaphase II until fertilization occurs. Disruptions in oocyte meiotic progression may lead to arrest at distinct developmental stages, thereby contributing to female infertility [3, 4].

RNA‐binding proteins (RBPs) are key regulators of RNA metabolism, modulating various post‐transcriptional processes, such as mRNA alternative splicing, stability, nuclear export, translation, and degradation [5]. During mammalian oocyte growth, chromatin remains in a decondensed, transcriptionally active state, enabling the production of mRNAs necessary for meiotic progression, fertilization, and the maternal‐to‐zygotic transition. However, as meiosis resumes prior to ovulation, chromatin undergoes condensation, resulting in transcriptional silencing from the late GV stage until the zygotic genome activation following fertilization [6]. Maternal mRNAs are indispensable for oocyte maturation and early embryonic development [7]. Notably, accumulating evidence suggests that RBPs play pivotal roles in regulating RNA metabolism during oocyte maturation and are essential for female fertility. For example, DAZL, a germ cell‐specific RBP, has been demonstrated to regulate oocyte maturation by either inhibiting or promoting the translation of maternal mRNA [8], and it is crucial for oocyte meiosis during embryonic development in humans [9]. A recent study revealed that another RBP, CPEB3, is essential for follicular development in mice; mutations in CPEB3 lead to downregulation of *Gdf9*, causing follicular development arrest and ultimately resulting in female subfertility [10]. Moreover, loss of the m^6^A reader protein YTHDC1 leads to widespread aberrant alternative splicing in the oocyte transcriptome, resulting in follicular development arrest and ultimately causing female infertility [11]. Therefore, the identification of key RBPs involved in oocyte maturation is of great importance, as it can enhance our understanding of the regulatory mechanisms underlying female fertility.

The heterogeneous nuclear ribonucleoproteins (hnRNPs), a highly conserved family, comprises over 20 RBP members ranging from hnRNPA to hnRNPU [12]. hnRNPs participate in various fundamental biological processes, including transcription, pre‐mRNA alternative splicing, mRNA stability, and translation [13]. Several hnRNPs are abundantly expressed in male germ cells [14], and play important roles in spermatogenesis [15, 16]. Our previous studies have demonstrated that hnRNPU interacts with two transcription factors, WT1 (Wilms' tumor 1) and SOX9 (SRY‐box transcription factor 9) to regulate *Sox8/9* transcription in Sertoli cells and modulates the alternative splicing of cell cycle‐related genes in spermatogonia [17, 18]. However, the role of hnRNPU in oocyte development remains unexplored.

In this study, we established two oocyte‐specific *Hnrnpu* knockout mouse models using *Gdf9*‐Cre and *Zp3*‐Cre to investigate the role of hnRNPU in female reproduction. Our results demonstrate that hnRNPU is indispensable for normal folliculogenesis and female fertility. Notably, deletion at earlier stages with *Gdf9*‐Cre led to a more profound defect in oocyte development compared to the later‐stage deletion driven by *Zp3*‐Cre, highlighting stage‐specific requirements for hnRNPU. Mechanistic studies, performed using the *Zp3*‐Cre model, showed that *Hnrnpu* loss in growing oocytes led to mitochondrial dysfunction, impaired oocyte‐granulosa cell communication, and increased apoptosis. Transcriptomic analysis demonstrated significant disruption in genes governing mitochondrial function and cell adhesion, with a substantial number of mitochondrial‐related transcripts displaying defective pre‐mRNA splicing. Taken together, our findings underscore the important role of hnRNPU in maintaining mitochondrial function and oocyte competence, thereby deepening our understanding of the molecular mechanisms contributing to female infertility.

## 2 Materials and Methods

### 2.1 Animals

Mice were housed at the specific pathogen‐free facility of Tongji Medical College under controlled conditions, including constant room temperature, controlled humidity, and a 12‐h light/dark cycle. Animals carrying floxed *Hnrnpu* gene variants were generously provided by Dr. Tom Maniatis's research group at Columbia University and were extensively characterized in a previous publication [17, 18]. The *Zp3*‐Cre (#003651) and *Gdf9*‐Cre (#011062) on the C57BL/6J genetic background were acquired from the Jackson Laboratory. To generate oocyte‐specific *Hnrnpu* knockout mice (*Zp3*‐Cre; *Hnrnpu* ^*flox/flox*^, referred to as ZcKO; *Gdf9*‐Cre; *Hnrnpu* ^*flox/flox*^, referred to as GcKO hereafter), *Hnrnpu* ^*flox/flox*^ female mice were crossed with *Zp3*‐Cre or *Gdf9*‐Cre male mice. The specific primer sequences used for genotyping are listed in Table S1.

### 2.2 Mouse Fecundity Test

To assess the fertility of *Hnrnpu* cKO female mice, wild‐type male mice (12‐week‐old) with proven fertility were mated with 8‐week‐old control and cKO female mice (*n* = 4 for each genotype) for six months. Cages were monitored daily, and the number of pups and litters was recorded.

### 2.3 Ovarian Histological Analysis

For the analysis of ovarian folliculogenesis, ovaries were collected from control and cKO female mice at 3 and 8 weeks of age. The ovaries were fixed in Bouin's solution (Sigma, HT10132) at 4°C overnight, washed with 75% ethanol, dehydrated through a graded ethanol series, embedded in paraffin (Leica, 39601095), and sectioned serially at a thickness of 5 μm. Sections were stained with hematoxylin–eosin (HE) Strain Kit (Solarbio, G1120). Every fifth section was analyzed to avoid repeated counting of the same follicle, and only follicles containing oocytes with a clearly visible nucleus were included in the analysis. Based on established criteria [19], follicles are classified into four developmental stages: primordial, primary, secondary, and antral. Briefly, primordial follicles are located near the ovarian cortex and consist of an oocyte surrounded by a single layer of flattened granulosa cells. Primary follicles contain an oocyte enclosed by a single layer of predominantly cuboidal granulosa cells. Secondary follicles are composed of an oocyte surrounded by multiple layers of cuboidal granulosa cells without an antrum. Antral follicles are characterized by an oocyte encircled by multiple layers of granulosa cells and a visible antral cavity. Follicle numbers at each stage were compared between groups.

### 2.4 Oocytes Collection and in Vitro Culture

To obtain germinal vesicle (GV) oocytes, 3‐week‐old female control and cKO mice were intraperitoneally injected with 5 IU of pregnant mare serum gonadotropin (PMSG; Sansheng, China). After 48 h, ovaries were obtained and punctured with a 1 mL syringe needle in M2 medium (Sigma, USA) supplemented with 10 μM milrinone (Millipore) to prevent GV meiotic resumption. For in vitro maturation, GV oocytes were washed in M2 medium without milrinone and then transferred to M16 medium (Sigma, USA) at 37°C in a 5% CO_2_ incubator. Following culture in M16 medium, oocytes were then collected at defined time points corresponding to different meiotic stages: 4 h (germinal vesicle breakdown, GVBD), 8 h (metaphase I, MI), and 16 h (metaphase II, MII). Oocytes at different stages were collected for subsequent analyses. For in vivo collection of MII oocytes, 8‐week‐old female control and cKO mice were injected with 5 IU PMSG, followed 48 h later by 5 IU of human chorionic gonadotrophin (hCG; Sansheng, China). Cumulus‐oocyte complexes were harvested from the ampullary region of the oviducts 16 h after hCG injection and treated with 0.5 mg/mL hyaluronidase (Sigma, USA) in M2 medium to remove surrounding granulosa cells and isolate MII oocytes.

### 2.5 Immunofluorescence Staining

Ovaries were fixed in 4% paraformaldehyde (PFA, Sigma, P6148) overnight at 4°C, followed by sequential dehydration in 5%, 10%, 12.5%, 15%, and 20% sucrose in PBS. Ovaries were then embedded in O.C.T. compound (Sakura Finetek, 4583) mixed with 10% sucrose (final composition: 50% O.C.T. and 10% sucrose) on dry ice. Cryosections of 5 μm thickness were prepared and washed three times with PBS. For antigen retrieval, sections were microwaved in 0.01 M sodium citrate buffer (pH 6.0) at medium power for 3 min, repeated three times until boiling. After cooling, sections were blocked with blocking buffer containing 5% normal donkey serum (Solarbio, SL050) and 5% fetal bovine serum (PAN‐Biotech, ST30‐3302) in 1% bovine serum albumin (BSA, BioFRoxx, 143064) for 1 h at room temperature (RT). Sections were then incubated overnight at 4°C with primary antibodies (listed in Table S2) diluted in the same blocking buffer. After washing with PBS, sections were incubated with appropriate secondary antibodies (listed in Table S2) in the dark for 1 h at RT. Nuclei were counterstained with Vectashield mounting medium containing DAPI (H1200, Vector Laboratories).

Immunofluorescent staining of oocytes was performed according to a published protocol [20]. Oocytes were fixed in 4% PFA for 30 min, permeabilized in 0.1% Triton X‐100 for 20 min, and blocked in 1% BSA for 1 h. The samples were then incubated with primary antibodies (listed in Table S2) overnight at 4°C, washed three times in 1 mg/mL polyvinyl pyrrolidone (PVP; Sigma) in PBS, followed by incubation with secondary antibodies (listed in Table S2) for 1 h at RT. For F‐actin staining, oocytes were washed three times after blocking and incubated for 1 h at room temperature with TRITC‐phalloidin (1:200; Abclonal, RM02835) in PBS containing 1% BSA. The samples were subsequently stained with DAPI. All images were captured using a FluoView 1000 microscope (Olympus, Japan) equipped with a digital camera (MSX2, Micro‐shot Technology Limited, China). Fluorescence intensities of N‐cadherin and β‐catenin were quantified in ImageJ using the plot profile tool, based on line‐scan analysis across the junction of oocytes and granulosa cells. The peaks in the profile were used for quantification of the relative fluorescence intensity.

### 2.6 TUNEL Assay

TUNEL staining was performed using the TUNEL Apoptosis Assay Kit (Beyotime, C1086) according to the manufacturer's instructions. Briefly, paraffin‐embedded ovarian sections were dewaxed in xylene and rehydrated through a graded ethanol series, followed by incubation in Tris–HCl buffer (100 mM, pH 7.5) containing 3% bovine serum albumin (BSA) and 20% fetal bovine serum (FBS) for 30 min at RT. Sections were then washed with PBS and incubated with the TUNEL reaction mixture for 1 h at 37°C. After staining nuclei with DAPI, fluorescence signals were visualized using a FluoView 1000 confocal laser scanning microscope (Olympus, Japan). The number of TUNEL‐positive cells was counted. All analyses were performed on multiple independent samples, and representative images are shown in the figures.

### 2.7 Mitochondria Staining

For mitochondria staining, oocytes were cultured in M2 medium containing 200 nM MitoTracker Red CMXRos (Beyotime, C1035) for 30 min at 37°C. After being washed three times with fresh M2 medium, oocytes were imaged using a FluoView 1000 confocal microscope (Olympus, Japan). Quantitative analysis of fluorescence intensity was performed with ImageJ software.

### 2.8 Detection of Mitochondrial Membrane Potential

Mitochondrial membrane potential in oocytes was assessed using the JC‐1 Mitochondrial Membrane Potential Assay Kit (Beyotime, C2006) according to the manufacturer's instructions. Oocytes were incubated with JC‐1 dye at 37°C under 5% CO_2_ for 20 min, followed by three washes with M2 medium. Images were immediately captured using a FluoView 1000 confocal microscope (Olympus, Japan). For each oocyte, mean fluorescence intensities of the red (JC‐1 aggregates) and green (JC‐1 monomers) channels were measured across the whole oocyte using ImageJ, and averaged for each group, which inherently normalizes for dye loading and oocyte size. The red to green fluorescence ratio was calculated. These red to green ratios for individual oocytes were subsequently averaged within each group and used to evaluate mitochondrial membrane potential.

### 2.9 Measurement of ROS Levels

Total intracellular ROS levels in live oocytes were assessed using the Reactive Oxygen Species Assay Kit (Beyotime, S0033S) following the manufacturer's protocol. Oocytes were incubated in M2 medium containing 10 μM DCFH‐DA at 37°C for 20 min. Mitochondrial ROS levels were determined by incubating oocytes with 5 μM MitoSOX Red (MCE, HY‐D1055) at 37°C for 30 min. Following incubation, oocytes were washed three times with M2 medium to remove excess dye. Fluorescence images were immediately captured using a FluoView 1000 confocal microscope (Olympus, Japan). Fluorescence intensity was quantified using ImageJ software.

### 2.10 Measurement of Oocyte ATP Content

The oocyte ATP level was measured using the Enhanced ATP Assay Kit (Beyotime, S0027) according to the manufacturer's instructions. Briefly, for each sample, 60 oocytes were added to 200 μL of ATP lysis buffer and mixed thoroughly by gently pipetting on ice. Then centrifuge at 12000 g for 5 min. 50 μL supernatant was added to 100 μL standard ATP working buffer. The ATP concentration was immediately measured using a plate reader (BioTek Synergy HTX). ATP concentrations were calculated from a standard curve and expressed per 60 oocytes.

### 2.11 RT‐PCR and Quantitative Real‐Time PCR (qRT‐PCR)

Total RNA was extracted from GV stage oocytes using TRIzol reagent (Invitrogen, 15596‐025) according to the manufacturer's recommended protocol. Briefly, GV oocytes were collected in TRIzol solution, homogenized by repeated pipetting, and incubated at room temperature for 5 min to allow phase separation. After chloroform (Sinopharm Chemical Reagent, H1445849) addition and centrifugation, the aqueous phase containing RNA was precipitated with isopropanol (Macklin, I811925), washed with 75% ethanol, and dissolved in RNase‐free water. The concentration and purity of RNA were assessed using a NanoDrop spectrophotometer (Thermo Fisher Scientific), with an A260/A280 ratio between 1.8 and 2.0. A total of 500 ng of RNA was reverse‐transcribed into cDNA using the High‐Capacity cDNA Reverse Transcription Kit (R211, Vazyme, Nanjing, China). RT‐PCR primers were designed within constitutive exons flanking the exon of interest. Standard PCR for analysis by gel electrophoresis was performed and visualized by running on a 2% agarose gel. Primer sequences used are found in Table S1. Uncropped gels are presented in Figure S5. Quantification of gel images was done by Image J software. Percentage spliced in values (PSI) were determined as the exon inclusion band intensity/(the exon inclusion band intensity + the exon exclusion band intensity) × 100. qRT‐PCR reactions were prepared using SYBR Green PCR Master Mix (Q711‐02, Vazyme, Nanjing, China) and gene‐specific primer pairs (sequences provided in Table S1) targeting transcripts of interest. Reactions were performed in technical triplicates on a Step One ABI real‐time PCR System (Applied Biosystems). Data were analyzed using the 2^−ΔΔCt^ method, with GAPDH serving as the endogenous control for normalization.

### 2.12 Single‐Cell RNA‐Seq and Bioinformatics Analysis

For each group (Control and ZcKO), three biological replicates were collected, with each replicate containing approximately 10–20 GV‐stage oocytes, which were pooled in lysis buffer containing RNase inhibitor (Beyotime, R0102). Single‐cell RNA‐seq libraries were generated following the Smart‐seq2 protocol [21, 22]. Briefly, mRNA was reverse transcribed using an oligo(dT) primer with a 5′ anchor sequence. During reverse transcription, a template‐switching oligo (TSO) was used to enable full‐length cDNA synthesis. The resulting cDNA was then amplified by PCR and purified using magnetic beads. Purified cDNA was quantified using QuantiFluor dsDNA System (Promega, E2670) and assessed for quality and fragment size using Qsep100 S2 Cartridge (BiOptic). Libraries were subsequently constructed using the Lifeint Transpose DNA Library Prep Kit for Illumina (catalog #A5005, China), following the manufacturer's instructions. Final libraries were quality‐checked and sequenced on the Illumina NovaSeq 6000 platform using paired‐end 150 bp reads (PE150). Raw sequencing reads were trimmed using Trim Galore to remove adapters and low‐quality bases. Clean reads were aligned to the mouse reference genome (mm10) using Hisat2. Differentially expressed genes (DEGs) analysis was performed using DESeq2 (V1.32.0). Genes with |log_2_FoldChange| > 1 and *p* < 0.05 were considered significantly differentially expressed. Gene Ontology (GO) enrichment analysis was performed using Cluster Profiler (v4.2.2). Alternative splicing (AS) events between control and *Hnrnpu* cKO oocytes were analyzed using rMATS software. Five major types of AS events were assessed: skipped exons (SE), retained introns (RI), mutually exclusive exons (MXE), alternative 5′ splice sites (A5SS), and alternative 3′ splice sites (A3SS). Differentially alternative splicing events were defined as those with a false discovery rate (FDR) < 0.05 and |ΔPSI| > 10% based on raw read counts. Single‐cell RNA‐seq data are deposited in the NCBI SRA (Sequence Read Achieve) database with the accession number PRJNA1377037.

### 2.13 ChIP Sequencing Data Analysis

Published mouse hnRNPU ChIP‐seq datasets and their corresponding input controls were obtained from GSE95116. For each data, raw sequencing reads were aligned to the mouse reference genome GRCm39 (UCSC mm39) (https://hgdownload.gi.ucsc.edu/goldenPath/mm39/bigZips/) using Bowtie (v1.3.0) with the parameters: ‐m 1 ‐n 2 ‐e 70 ‐k 1 ‐‐best ‐l 75. PCR duplicates were removed using Picard. ChIP‐seq peak calling was performed with MACS2 (v2.2.5) in broad‐peak mode, using the input samples as controls and an FDR threshold of < 0.01. Coverage tracks were normalized to reads per million (RPM), and biological replicates were merged for downstream analyses. Heatmaps and metagene profiles of hnRNPU enrichment across ±2 kb of transcription start sites (TSS) were generated using deepTools (v3.5.2). ChIP‐Seq signal tracks were visualized in the Integrative Genomics Viewer (IGV) browser.

### 2.14 Exon Ontology Analysis

Alternatively spliced cassette exons identified by rMATS were used for exon ontology analysis. Genomic coordinates based on the mouse reference genome mm39 were converted to the human hg19 assembly using the UCSC LiftOver tool with a minimum remapping ratio of 0.8 (https://genome.ucsc.edu/cgi‐bin/hgLiftOver). Exons that were successfully transferred were subsequently analyzed using the Exon Ontology platform [23] to annotate protein features associated with alternative splicing (https://fasterdb.ens‐lyon.fr/ExonOntology/). Annotated exon‐associated features were deduplicated and summarized by gene and feature category, and gene‐feature category associations were visualized using a Sankey diagram generated in R (networkD3 package).

### 2.15 Statistical Analysis

All quantitative data were presented as mean ± SEM. Statistical analysis of differences between groups was conducted using one‐way ANOVA or Student's *t*‐test with SPSS19.0 software. *p*‐values were indicated in figures as *p < 0.05; **p < 0.01; ***p < 0.001; and ****p < 0.0001. Data quantitation was processed by GraphPad Prism software. Graphical abstract was created with BioRender.com.

## 3 Results

### 3.1 hnRNPU Exhibits Dynamic Localization During Oocyte Development and Early Embryogenesis

**FIGURE 1:**
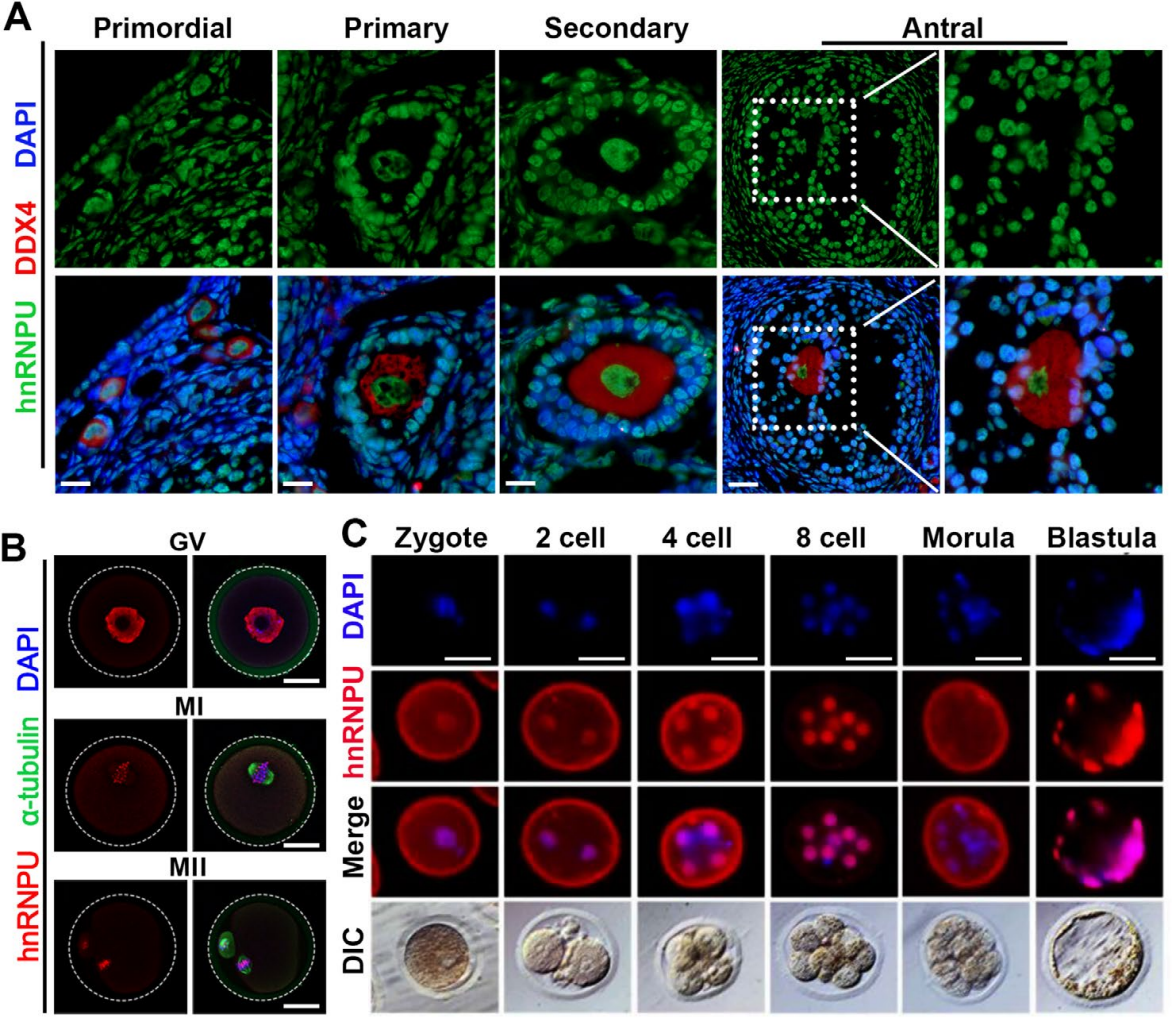
The expression pattern of hnRNPU during oocyte development and early embryonic development. (A) Co‐immunofluorescent staining of hnRNPU (green) and DDX4 (germ cell marker, red) in ovarian sections at different follicles stages (Primordial, Primary, Secondary, and Antral follicles). Nuclei were stained with DAPI. Scale bar = 50 μm. (B) Co‐immunofluorescent staining of hnRNPU (red) and α‐tubulin (spindle marker, green) in oocytes at different developmental stages (GV, MI and MII). Nuclei were stained with DAPI. Scale bar = 50 μm. (C) Immunofluorescent staining of hnRNPU (red) in early‐stage mouse embryos (Zygote, 2 cell, 4 cell, 8 cell, Morula and Blastula). Nuclei were stained with DAPI. Scale bar = 50 μm.

To investigate the role of hnRNPU in oocyte development, we first examined its precise cellular localization in the ovary. Immunofluorescence (IF) analysis of ovarian tissues revealed that hnRNPU is predominantly localized to the nuclei of oocytes in primordial, primary, secondary, and antral follicles (Figure 1A). Since oocyte development is accompanied by extensive mRNA synthesis during follicular development [24], hnRNPU may play a potential role in regulating mRNA synthesis during this process. To further elucidate the distribution of hnRNPU during oocyte development, we performed IF staining on isolated GV oocytes. hnRNPU was predominantly localized to the nuclei of GV oocytes (Figure 1B). Interestingly, as oocytes matured to the metaphase I (MI) and metaphase II (MII) stages, hnRNPU became localized to the chromosome region (Figure 1B). In addition, hnRNPU was detected in the nuclei of early embryos at various developmental stages (Figure 1C). These findings suggest that hnRNPU displays dynamic localization during oocyte development and early embryogenesis, supporting its potential involvement in these critical developmental stages.

### 3.2 Oocyte‐Specific Ablation of *Hnrnpu* Impairs Follicular Development and Causes Female Infertility

**FIGURE 2:**
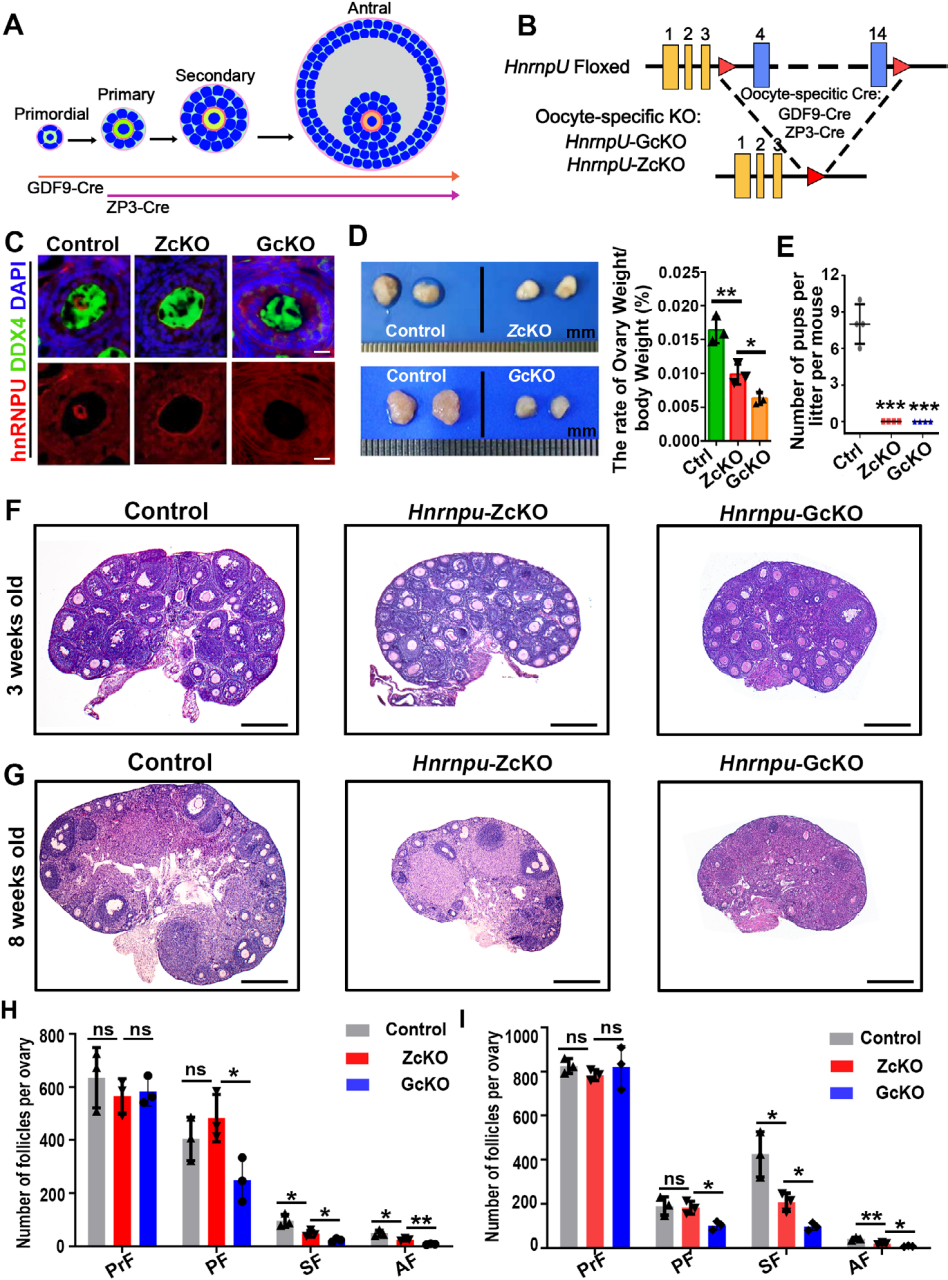
Oocyte‐specific knockout of hnRNPU leads to aberrant folliculogenesis and female infertility. (A) Schematic diagram showing the different stage of follicles. *Zp3*‐Cre is active in oocytes of primary follicles; *Gdf9*‐Cre is active in oocytes of primordial follicles. (B) Schematic diagram showing the targeting strategy of generating a floxed *Hnrnpu* allele through homologous recombination in oocytes. Red triangles represent loxP cassettes. Exons 4 to 14 will be deleted after *Zp3*‐Cre and *Gdf9*‐Cre mediated recombination. (C) IF staining of hnRNPU in ovarian sections from adult control, ZcKO and GcKO mice. DDX4 was co‐stained to mark oocyte. The DNA was stained with DAPI. Scale bar = 50 μm. (D) Gross morphology of ovaries harvested from control, ZcKO and GcKO mice at 8 weeks old. Quantification of the ovary‐to‐body weight ratio is shown on the right. Data are presented as mean ± SEM (*n* = 3 for each genotype), *p < 0.05, **p < 0.01. (E) Litter size analysis of control, ZcKO and GcKO mice. Control, ZcKO and GcKO females were crossed with WT males for 6 months. ZcKO and GcKO female mice failed to produce any pups. Data are presented as Mean ± SEM (*n* = 4 for each group), ***p < 0.001. (F) Hematoxylin and eosin (H&E) staining of ovarian paraffin sections from 3‐week‐old Control, ZcKO and GcKO mice. Scale bar = 500 μm. (G) H&E staining of ovarian paraffin sections from 8‐week‐old Control, ZcKO and GcKO mice. Scale bar = 500 μm. (H) Quantitative analysis of follicle numbers at different stages in ovarian sections from 3‐week‐old Control, ZcKO and GcKO mice. Data are presented as Mean ± SEM (*n* = 3 biological replicates for each group), *p < 0.05, **p < 0.01. (I) Quantitative analysis of follicle numbers at different stages in ovarian sections from 8‐week‐old Control, ZcKO and GcKO mice. Data are presented as Mean ± SEM (*n* = 3 biological replicates for each group), *p < 0.05, **p < 0.01.

To investigate the physiological role of hnRNPU in oocyte development, we generated two oocyte‐specific *Hnrnpu* knockout mouse models by crossing *Hnrnpu* ^*flox/flox*^ mice with *Zp3*‐Cre and *Gdf9‐Cre* transgenic lines, respectively. *Zp3‐Cre* is active in oocytes of primary follicles after postnatal Day 5, while *Gdf9‐Cre* is active in oocytes of primordial follicles starting from postnatal Day 3 (Figure 2A). The floxed *Hnrnpu* allele contains loxP sites flanking exons 4–14, allowing Cre‐mediated recombination to generate a null allele at the respective stages of follicle development (Figure 2B). Mice with genotype *Hnrnpu* ^*flox/Del*^ *; Zp3*‐Cre are referred to as ZcKO, and those with *Hnrnpu* ^*flox/Del*^ *; Gdf9*‐Cre are referred to as GcKO. Age‐matched *Hnrnpu* ^*flox/flox*^ or *Hnrnu* ^*flox/+*^ mice serve as controls. Co‐immunostaining for hnRNPU and the germ cell marker DDX4 in ovarian sections confirmed the specific deletion of *Hnrnpu* in ZcKO and GcKO mice (Figure 2C). Morphological analysis revealed that the ovaries of adult ZcKO females were significantly smaller than those of control mice, and GcKO ovaries were even smaller than those of ZcKO mice (Figure 2D). To assess fertility, both *Hnrnpu* cKO female mice were mated with proven fertile wild‐type (WT) males for 6 months. Neither ZcKO nor GcKO females produced any offspring, indicating complete infertility in both models (Figure 2E). To explore the underlying cause of infertility, we examined follicular development at different stages. Histological analysis of ovaries from 3‐week‐old mice revealed a marked reduction in secondary and antral follicles in ZcKO mice compared to controls. In GcKO ovaries, follicle numbers were significantly decreased starting from the primary stage (Figure 2F,H). Furthermore, in 8‐week‐old ZcKO ovaries, the transition from secondary to antral follicles was severely impaired, with a substantial decrease in the number of antral follicles. In contrast, antral follicles were completely absent in GcKO ovaries (Figure 2G,I). These findings indicate that the loss of hnRNPU leads to arrested follicular development. Moreover, the earlier depletion of hnRNPU in oocytes, as in the GcKO model, leads to more severe ovarian defects. Together, these results highlight the essential role of hnRNPU in regulating folliculogenesis and female fertility.

### 3.3 Oocyte‐Specific Knockout of *Hnrnpu* Causes Defects in Oocyte Meiotic Maturation

**FIGURE 3:**
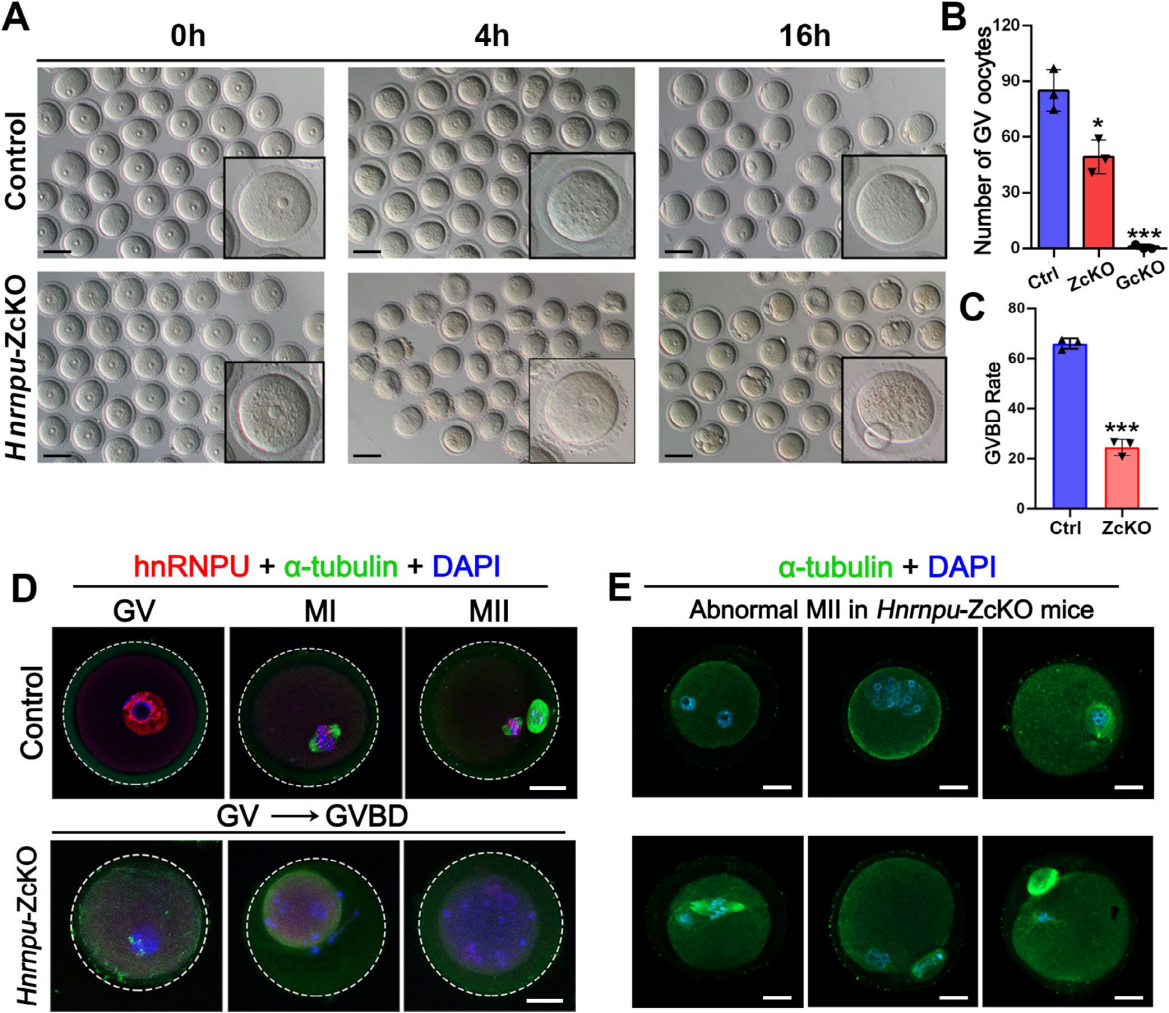
Deletion of *Hnrnpu* specifically in oocytes leads to defects in oocyte meiotic progression. (A) The gross morphology of oocytes collected at the time points as indicated for GV (0 h), and cultured in vitro for GVBD (4 h) and MII (16 h) from PMSG‐primed 3 weeks old control and ZcKO mice. Due to the inability to retrieve enough GV oocytes for in vitro culture from PMSG‐primed GcKO mice, representative images for this group are not included. Scale bar = 100 μm. (B) Quantitative analysis of GV oocytes from PMSG‐primed 3 weeks old control, ZcKO and GcKO mice. Data were presented as mean ± SEM, *n* = 3. *p < 0.05, ***p < 0.001 by two‐tailed Student's t‐test. (C) Quantitative analysis of GVBD‐stage oocytes derived from control and ZcKO GV oocytes after 4 h of in vitro culture. Data were presented as mean ± SEM, *n* = 3. ***p < 0.001 by two‐tailed Student's t‐test. (D) Co‐immunofluorescent staining of hnRNPU (red) with α‐tubulin (green) in control and ZcKO oocytes at different culture time points (0 h‐GV, 8 h‐MI, and 16 h‐MII). Nuclei were stained with DAPI. Scale bar = 20 μm. (E) IF staining of α‐tubulin (green) in ZcKO abnormal oocytes cultured 16 h in vitro. Oocytes showing disrupted spindle structure and misaligned chromosomes. Nuclei were stained with DAPI. Scale bar = 20 μm.

To further elucidate the effects of *Hnrnpu* knockout on follicular development, we focused on the process of oocyte meiotic maturation. Superovulation was induced in control, ZcKO and GcKO mice by PMSG injection, followed 48 h later by hCG administration. Ovulation was then assessed 16 h after hCG injection. We found that neither ZcKO mice nor GcKO mice were able to produce mature MII oocytes; interestingly, a few morphologically abnormal and immature oocytes were retrieved from the oviduct ampulla of ZcKO mice (Figure S1A–C). In addition, IF staining of these ZcKO oocytes displayed abnormal spindle formation, misaligned chromosomes and failed to form a proper actin cap, ultimately resulting in impaired oocyte maturation (Figure S1D,E). To further characterize the observed developmental blockade, we isolated GV stage oocytes from control, ZcKO and GcKO mice following PMSG injection and cultured them in vitro. A significant reduction in the number of GV oocytes was observed in ZcKO mice, and GV oocytes were nearly absent in GcKO mice (Figure 3A,B and Figure S2). Therefore, subsequent studies were performed using ZcKO oocytes. After 4 h culture in M16 medium, we found that most ZcKO oocytes failed to resume meiosis normally, as shown by a dramatically reduced rate of germinal vesicles breakdown (GVBD) (Figure 3A,C). Meanwhile, we found that ZcKO oocytes were unable to progress to the MI and MII stages, as evidenced by the absence of spindle formation and failure to extrude the first polar body (Figure 3A,D), which is aligned with the in vivo findings (Figure S1C–E). To further determine the effects of *Hnrnpu* deletion on oocyte meiotic divisions, ZcKO oocytes cultured in vitro for 16 h were co‐staining with α‐tubulin (a cytoskeleton marker) and DAPl. This analysis revealed various abnormalities in ZcKO oocytes, including disrupted spindle assembly, misaligned chromosomes, failure of polar body extrusion, and aberrant polar body morphology (Figure 3E). Taken together, these results suggest that hnRNPU is essential for oocyte meiotic progression. Deletion of hnRNPU in oocytes using *Zp3*‐Cre leads to meiotic arrest primarily at the GV stage. In contrast, deletion of hnRNPU in oocytes using *Gdf9*‐Cre results in even more severe meiotic defects, likely causing arrest at an earlier stage of oocyte development.

### 3.4 Oocyte‐Specific Knockout of *Hnrnpu* Disrupts Maternal Transcriptome and Alternative Splicing of Mitochondria‐Related Genes

**FIGURE 4:**
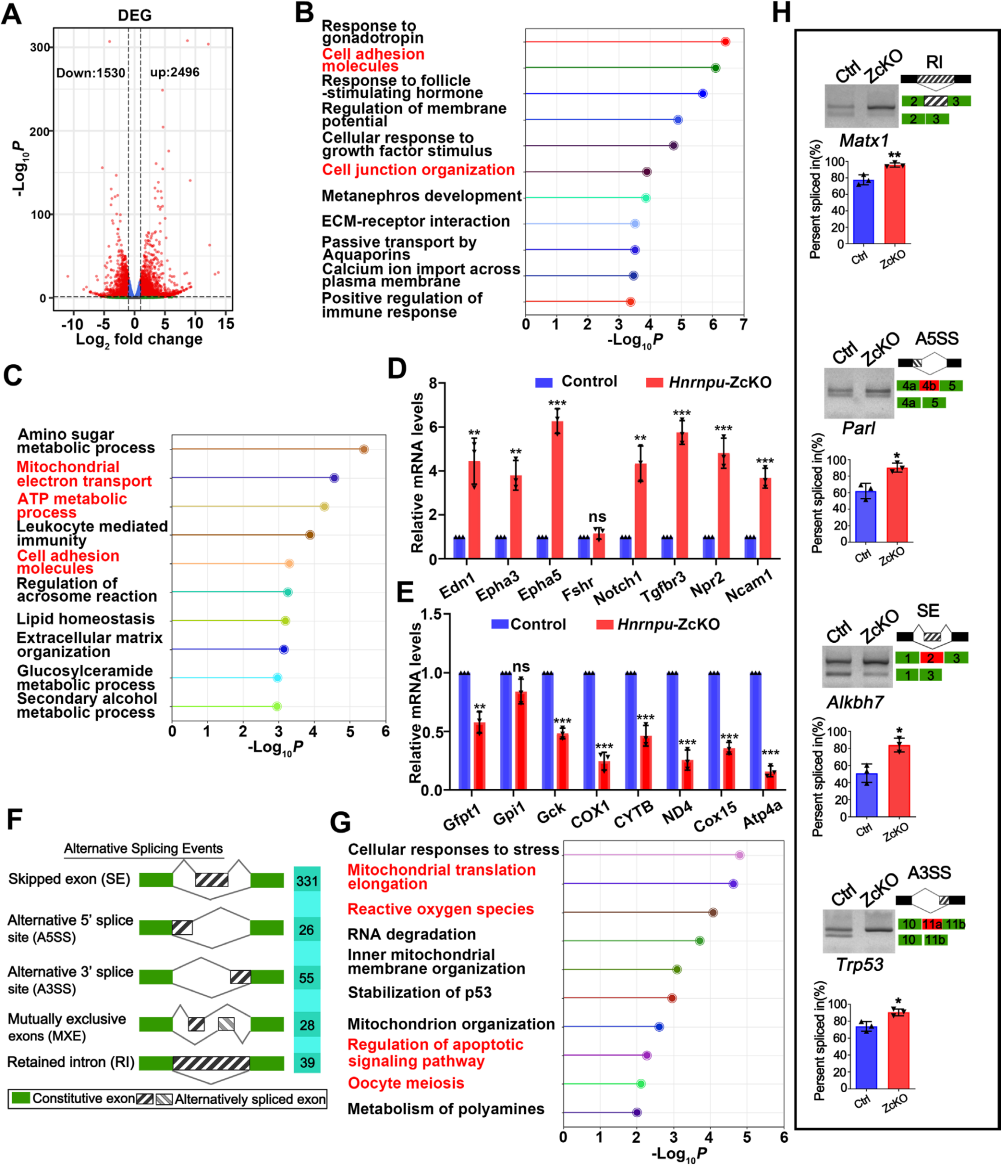
hnRNPU regulates the maternal transcriptome and modulates alternative splicing of mitochondria‐related genes in oocytes. (A) Volcano plot showing differentially expressed genes (DEGs) in GV oocytes from ZcKO and control mice. Left red color: Down‐regulated; Right red color: Up‐regulated; Cutoff: Fold change (FC) ≥ 2, adjusted *p* < 0.05. (B, C) GO enrichment analysis for the upregulated (B) and downregulated DEGs (C) in ZcKO oocytes compared to control. (D, E) Real‐time qPCR validation of selected upregulated (D) and downregulated (E) genes in ZcKO GV oocytes. Data are presented as mean ± SEM, *n* = 3 biological replicates. Statistical test is two‐tailed Student's t‐test. **p < 0.01, ***p < 0.001. (F) Five types of AS events significantly affected in ZcKO oocyte, with the number of AS events indicated. (G) GO enrichment analysis of genes exhibiting abnormal AS events in ZcKO oocytes. (H) RT‐PCR validation of four selected mitochondrial‐associated genes (*Mtx1*, *Alkbh7*, *Trp53*, and *Parl*) in control and ZcKO GV oocytes. On the right is a schematic representation of alternatively spliced exons and the percentage of exons included (PSI) in control and ZcKO oocytes. Data were obtained from three biologically independent mice per genotype. *p < 0.05, **p < 0.01.

Oocyte growth and meiotic progression require precise regulation of RNA transcription and degradation [25, 26]. Transcription is highly active in growing oocytes, reaching its peak at the early stage of antral follicles [27]. To gain insights into the mechanisms underlying *Hnrnpu*‐dependent oocyte growth, we isolated GV‐stage oocytes from the ovarian of control and ZcKO mice, as oocytes were nearly virtually absent in GcKO mice. We then performed single‐cell RNA sequencing using the Smart‐seq2 protocol to compare their transcriptomic profiles. As expected, we identified a large number of differentially expressed genes (DEGs) between control and ZcKO oocytes, with 2496 genes significantly upregulated and 1530 genes significantly downregulated in ZcKO oocytes (Figure 4A, Table S3). Notably, Gene Ontology (GO) enrichment analyses revealed that the significantly upregulated genes were mainly enriched in cell adhesion molecules, cell junction organization, and response to follicle‐stimulating hormone, while the downregulated genes were mainly associated with ATP metabolism processes and regulation of mitochondrial function (Figure 4B,C). To validate the reliability of our sequencing data, we performed qRT‐PCR analysis on a subset of DEGs associated with follicular development and mitochondrial function. The results showed that *Notch1, Edn1*, and *Epha3* were upregulated, while *CYTB*, *ND4, COX15* and *COX1* were downregulated in ZcKO oocytes, consistent with the RNA sequencing data (Figure 4D,E). Thus, our findings indicate that the maternal transcriptome is disrupted in ZcKO oocytes at the GV stage.

Given that many RNA‐binding proteins also bind DNA to modulate gene transcription, we re‐analyzed a published hnRNPU ChIP‐seq dataset from mouse hepatocytes (GSE95116) [28] and overlapped the hnRNPU peaks with our Smart‐seq2 DEGs. We observed a strong enrichment of hnRNPU binding at the TSS of our DEGs when compared to the input control (Figure S3A and Table S4). This suggests that hnRNPU is likely to regulate a substantial portion of these genes at the transcriptional level. Venn analysis further showed that 554 upregulated genes and 408 downregulated genes overlap with hnRNPU‐bound genes (Figure S3B). indicating hnRNPU can influence both gene activation and repression. Functional enrichment analysis of up‐regulated genes revealed enrichment in response to “Regulation of cell apoptotic process”, “TGF‐beta signaling pathway”, and “Regulation of DNA‐binding transcription factor activity”. Down‐regulated genes were enriched in “Glucose catabolic process”, “Lysosome organization”, “Regulation of cell adhesion” (Figure S3C). Four representative genes (*Gfpt1*, *Cox15*, *Zfp36l2*, and *Cdc5l*) closely linked to oocyte development show clear hnRNPU binding peaks across all promoters of their loci (Figure S3D), indicating that hnRNPU may function as a critical transcriptional regulator to control the expression of genes required for oogenesis.

Additionally, hnRNPU is a well‐established RNA‐binding protein that has been widely implicated in the regulation of alternative splicing (AS) in various cells, such as cardiomyocytes and gastric cancer cells [29, 30, 31]. To investigate whether hnRNPU influences AS events in oocytes, we performed alternative splicing analysis based on RNA sequencing data. A total of 479 aberrant AS events were identified, with skipped exon (SE) events constituting the majority (69.1%, 331 out of 479) (Figure 4F, Table S5). Other AS events included alternative 3′ splice sites (A3SS, 55 events), alternative 5′ splice sites (A5SS, 26 events), mutually exclusive exons (MXE, 28 events), and retained introns (RI, 39 events). Among them, only 12 genes exhibited significant differences in both mRNA abundance and splicing (Figure S4A). Of note, we found that 303 hnRNPU‐regulated exons were CDS‐mapped and were further classified based on whether they were open reading frame (ORF)‐preserving (i.e., exon length is a multiple of 3). Among these, 153 hnRNPU‐regulated exons preserve the ORF (Figure S4B), and none were predicted to undergo nonsense‐mediated RNA decay. This result is consistent with our transcriptome data, in which the majority of mRNAs harboring hnRNPU‐regulated exons in oocytes do not exhibit a significant change in their overall abundance (Figure S4A). On the contrary, these exons are likely to alter the intrinsic structure and function of the encoded proteins. To further probe the functional properties of these exons, we performed exon ontology analysis, which revealed significant enrichment of sequences encoding molecular regions associated with post‐translational modifications (PTMs), structure, cellular localization, binding, and catalytic activities (Figure S4C).

GO enrichment analysis further indicated that genes affected by abnormal AS events were mainly associated with mitochondrial function regulation, apoptosis signaling pathway, and oocyte meiosis (Figure 4G). Subsequently, we selected four mitochondrial function‐related genes (*Mtx1*, *Alkbh7*, *Trp53*, and *Parl*) for further validation. RT‐PCR confirmed that *Mtx1*, *Alkbh7*, *Trp53*, and *Parl* underwent abnormal alternative splicing, consistent with the RNA sequencing results (Figure 4H). Most notably, P53 encoded by the *Trp53* gene is a core factor in the apoptotic pathway, and its expression has been reported to be regulated by hnRNPU [32]. In hnRNPU cKO oocytes, abnormal *Trp53* splicing generates longer transcripts with extended 3′UTRs (Figure 4H and Figure S4D); given the established role of 3′UTR length in promoting translation [33], this likely explains the significant increase in P53 that we observed in the mutant ovary via immunofluorescence (Figure S4E). Thus, hnRNPU loss promotes P53 translation through splicing‐mediated 3′UTR elongation. Intriguingly, the aberrant splicing of other apoptosis‐related genes is also likely to result in functional changes to their respective proteins (Figure S4F).

Taken together, our results indicate that hnRNPU is essential for maintaining the maternal transcriptome and further suggest its involvement in the alternative splicing regulation of genes required for oocyte maturation.

### 3.5 Oocyte‐Specific Knockout of *Hnrnpu* Leads to Abnormal Mitochondrial Function in Oocytes

**FIGURE 5:**
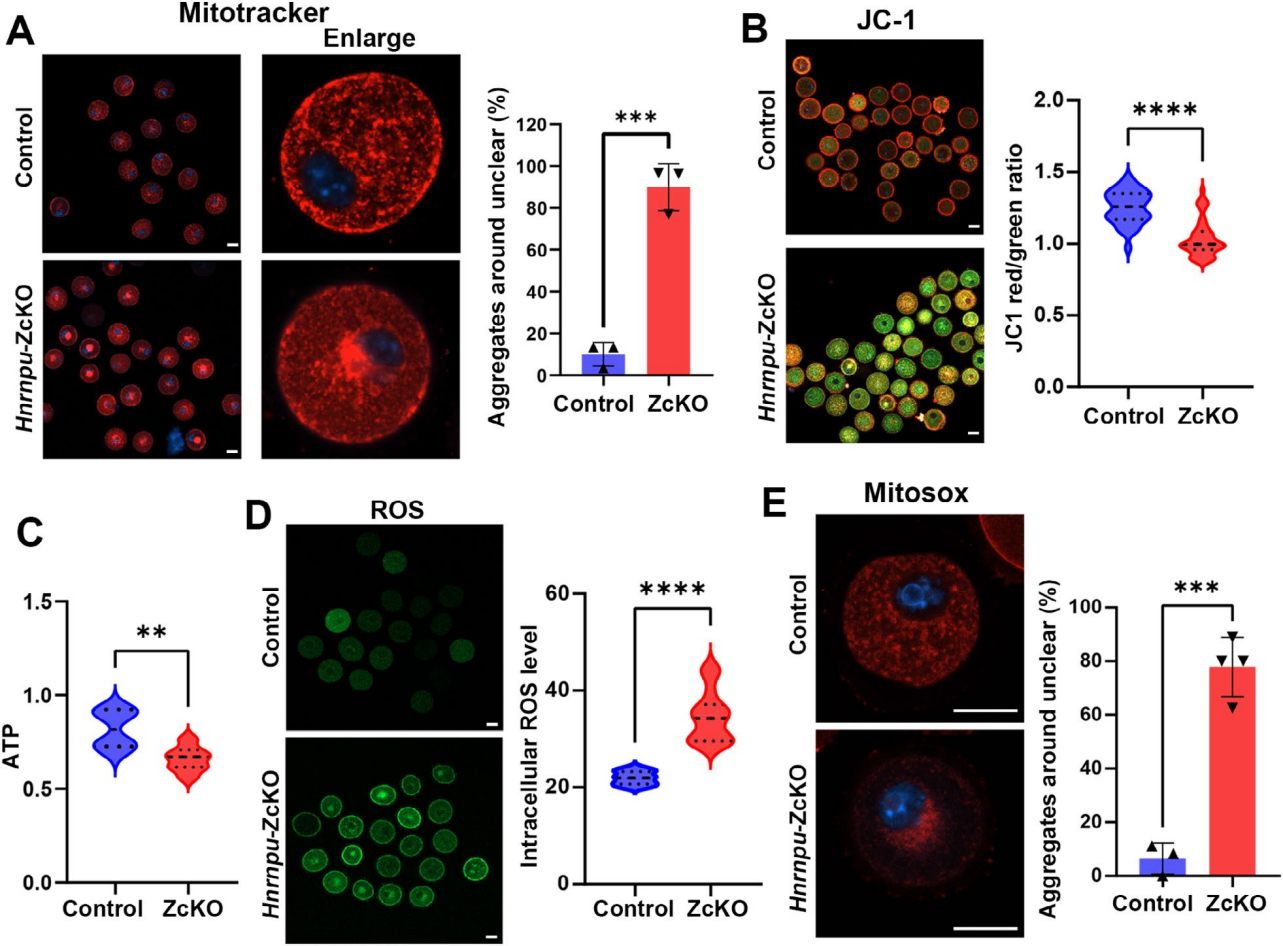
Mitochondrial dysfunction in ZcKO oocytes. (A) The control and ZcKO GV oocytes were labeled with Mito‐Tracker Red to visualize mitochondrial localization (left panel). Quantification of GV oocytes exhibiting mitochondrial aggregation around the nucleus was shown in right panel (*n* = 3 biological replicates for each group). Data are presented as Mean ± SEM, ***p < 0.001. Scale bar = 30 μm. (B) Representative images showing mitochondrial membrane potential in control and ZcKO oocytes assessed by JC‐1 staining (left panel). Green fluorescence indicates inactive mitochondria; red indicates active mitochondria. Scale bar = 30 μm. Quantification of mitochondrial membrane potential is represented by the JC‐1 red/green fluorescence ratio (right panel, *n* = 32 for control and 46 for ZcKO from three biological replicates, Data are presented as Mean ± SEM, ****p < 0.0001). (C) The graph of ATP levels in control and ZcKO GV oocytes. Data are presented as means ± SEM (*n* = 6 biological replicates). Two‐tailed student's t‐test was used to calculate *p* values. **p < 0.01. (D) Intracellular ROS levels were measured using DCFH‐DA staining in control and ZcKO GV oocytes (left panel). Scale bar = 10 μm. Quantification is shown in the right panel. Data are presented as Mean ± SEM, *n* = 16 for control and 20 GV oocytes for ZcKO from three biological replicates, ****p < 0.0001. (E) Mitochondrial ROS levels stained using MitoSOX Red in control and ZcKO GV oocytes (left panel). Scale bar = 10 μm. Quantification of perinuclear mitochondrial ROS levels is shown in the right panel. Data are presented as Mean ± SEM, *n* = 3 biological replicates for control and 4 biological replicates for ZcKO, ***p < 0.001.

Mitochondria are critical for supporting oocyte development. Our single‐cell RNA sequencing analysis revealed transcriptional dysregulation of genes related to mitochondrial function in ZcKO oocytes (Figure 4C). AS analysis also showed that hnRNPU regulates the AS of mitochondrial‐associated transcripts (Figure 4G,H). To evaluate whether mitochondrial function was affected, we first examined mitochondrial distribution in control and ZcKO GV oocytes. During oocyte growth, mitochondria transition from a dispersed cytoplasmic pattern in growing oocytes to a perinuclear localization prior to meiotic resumption [34]. In ZcKO GV oocytes, we observed a significantly higher proportion of mitochondria with perinuclear localization compared to controls (Figure 5A). We next assessed mitochondrial membrane potential, as it is an important indicator of mitochondrial activity during oocyte maturation [35, 36]. Using the fluorescent dye JC‐1, which shifts from green to red as mitochondrial membrane potential increases, we found a significant reduction in red fluorescence in ZcKO oocytes, indicating mitochondrial depolarization (Figure 5B). Consistently, ATP levels were also significantly decreased, indicating impaired mitochondrial bioenergetics (Figure 5C). As mitochondrial dysfunction is linked to reactive oxygen species (ROS) accumulation [37], we measured both intracellular ROS levels and mitochondrial ROS levels and observed a marked increase in ZcKO oocytes, reflecting elevated oxidative stress (Figures 5D,E). Taken together, these findings suggest that *Hnrnpu* is essential for maintaining mitochondrial functions. Its deletion disrupts mitochondrial function by inducing mitochondria aggregation, membrane depolarization, ATP depletion, and ROS accumulation, finally impairing oocyte maturation.

### 3.6 Loss of *Hnrnpu* in Oocytes Impairs Oocyte‐Granulosa Cell Crosstalk and Induces Follicular Apoptosis

**FIGURE 6:**
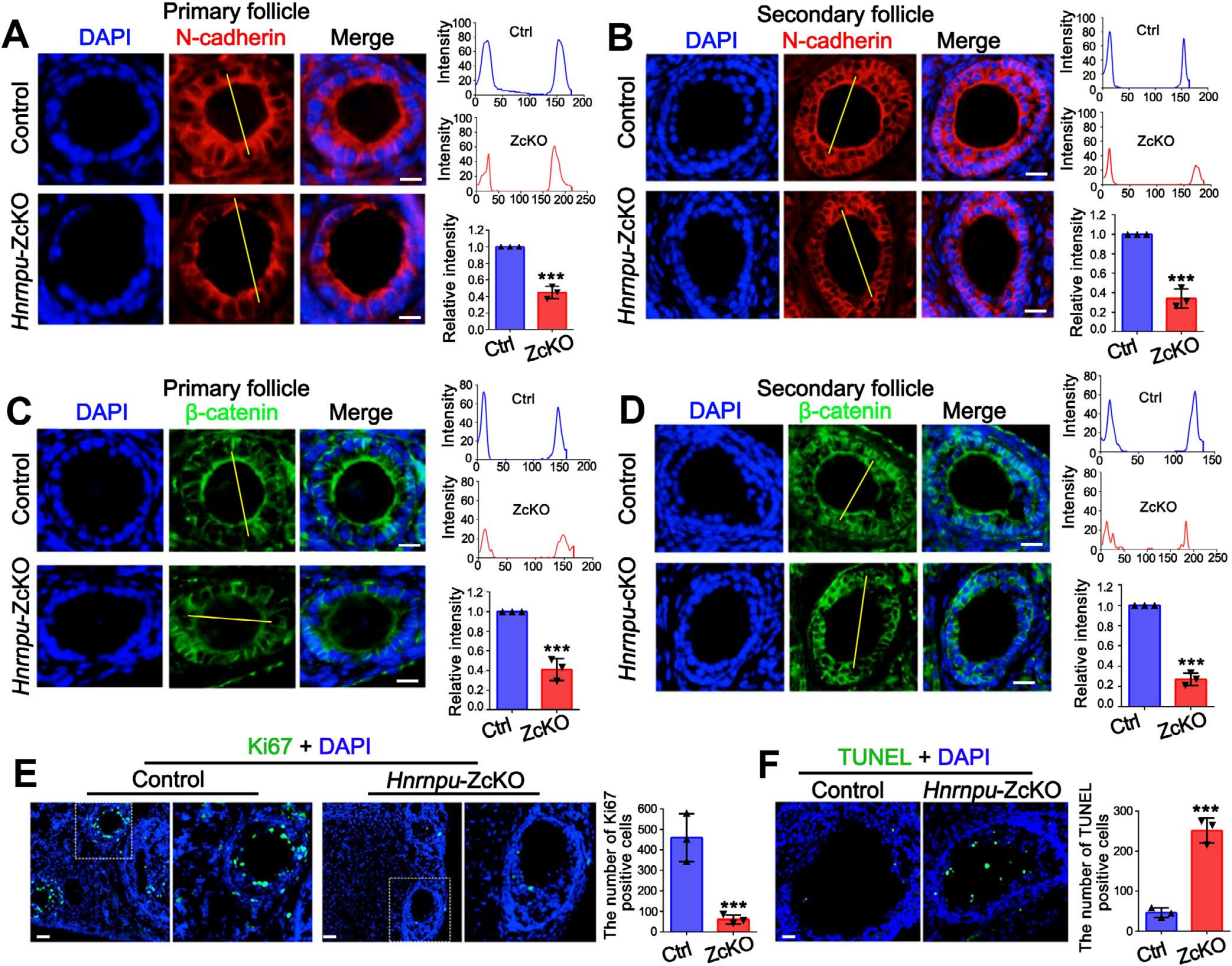
hnRNPU is essential for oocyte‐granulosa cell communication and granulosa cell proliferation. (A) Immunofluorescent staining (IF) staining of N‐cadherin (red) in primary follicles of control and ZcKO ovaries. Nuclei were stained with DAPI. Scale bar = 100 μm. Quantification of relative fluorescence intensity of N‐cadherin at the junction of oocytes and granulosa cells based on line‐scan profiles is shown in the right panel (*n* = 3 biological replicates; mean ± SEM; ***p < 0.001). (B) IF staining of N‐cadherin (red) in secondary follicles of control and ZcKO ovaries. Nuclei were stained with DAPI. Scale bar = 100 μm. Quantification of relative fluorescence intensity of N‐cadherin at the junction of oocytes and granulosa cells is shown in the right panel (*n* = 3 biological replicates; mean ± SEM; ***p < 0.001). (C) IF staining of β‐catenin (green) in primary follicles of control and ZcKO ovaries. Nuclei were stained with DAPI. Scale bar = 100 μm. Quantification of relative fluorescence intensity of β‐catenin at the junction of oocytes and granulosa cells is shown in the right panel (*n* = 3 biological replicates; mean ± SEM; ***p < 0.001). (D) IF staining of β‐catenin (green) in secondary follicles of control and ZcKO ovaries. Nuclei were stained with DAPI. Scale bars = 100 μm relative fluorescence intensity of β‐catenin at the junction of oocytes and granulosa cells. (E) IF staining of Ki67 (proliferation marker, green) in adult control and ZcKO ovaries. Nuclei were stained with DAPI. Scale bar = 50 μm. Quantification of Ki67 positive cells of ovary sections is shown in the right panel (*n* = 3 biological replicates; mean ± SEM; ***p < 0.001). (F) Apoptotic cell death in the ovaries of adult control and ZcKO mice was evaluated by TUNEL assay. Scale bar = 50 μm. Quantification of TUNEL positive cells of ovary sections is shown in the right panel (*n* = 3 biological replicates; mean ± SEM; ***p < 0.001).

Given the crucial role of bidirectional communication between oocytes and granulosa cells (GCs) in folliculogenesis, and based on our single‐cell RNA sequencing data showing that numerous dysregulated genes in ZcKO oocytes were enriched in pathways related to cell adhesion and cell junction organization, we next investigated whether the intercellular communication between oocyte and granulosa cell was disrupted. Transzonal projections (TZPs), cytoplasmic extensions from GCs that penetrate the zona pellucida to contact the oocyte, are essential for this communication and are regulated by cell adhesion molecules such as N‐cadherin and β‐catenin [38]. Immunofluorescence staining of N‐cadherin and β‐catenin in the primary and secondary follicles of ZcKO ovaries revealed a dramatic decrease compared to controls (Figure 6A–D). In addition, ZcKO ovaries exhibited reduced GC proliferation, as shown by staining of Ki67, a well‐established marker of cell proliferation [39, 40]. This reduction was further confirmed by quantitative analysis of Ki67 positive GCs (Figure 6E). TUNEL assay showed significantly increased follicular apoptosis in ZcKO ovaries (Figure 6F), consistent with our single‐cell RNA sequencing data, which revealed abnormal alternative splicing of genes involved in the apoptotic signaling pathway (Figure 4G and Figure S4D,E). Collectively, these findings indicate that hnRNPU may play a critical role in regulating communication between oocytes and surrounding granulosa cells (GCs). Loss of hnRNPU in oocytes disrupts this bidirectional signaling, impairs GC function, induces follicular apoptosis, and ultimately leads to defective folliculogenesis and female infertility.

## 4 Discussion

Oocyte maturation is a critical process during which the maternally inherited transcriptome must be properly regulated to enable oocytes to acquire developmental competence. Increasing evidence suggests that RBPs govern the shaping of the maternal transcriptome by regulating alternative splicing. For example, SRSF1 directly regulates the alternative splicing of premature ovarian insufficiency (POI)‐related genes in oocytes [41], whereas SRSF3 maintains transcriptome integrity through alternative splicing regulation [42]. Other RBPs, such as BCAS2 [43], YTHDC1 [11] and ESRP1 [4], are also involved in alternative splicing regulation in oocytes. In our previous studies, we demonstrated that RNA‐binding protein hnRNPU plays a pivotal role in spermatogenesis. In mouse Sertoli cells, hnRNPU regulates the expression of the transcription factors Sox8/9 [17], while in male germ cells, it modulates the alternative splicing of cell cycle‐related genes [18]. Interestingly, our current study identified a widespread dysregulation in pre‐mRNA splicing of mitochondrial function‐related genes in *Hnrnpu*‐deficient oocytes. hnRNPU exhibited a dynamic expression pattern throughout oocyte development. Oocyte‐specific deletion of *Hnrnpu* caused distinct phenotypes depending on the timing of deletion. *Zp3*‐Cre‐mediated deletion caused oocyte arrest at the germinal vesicle (GV) stage, whereas *Gdf9*‐Cre‐mediated deletion severely impaired the retrieval of GV oocytes, indicating a more severe developmental arrest. Our single‐cell RNA‐seq analysis revealed extensive aberrant maternal transcripts and alternative splicing of mitochondrial function‐related genes. These findings highlight a critical role for hnRNPU in regulating alternative splicing during oocyte development.

Oocyte growth and maturation are energy‐consuming processes and mitochondria provide the main source for ATP production [44, 45]. Multiple genes have been identified as key factors for mitochondrial function. For instance, Metaxin‐1 (MTX1) is a mitochondrial outer membrane protein that interacts directly with its partner MTX2 [46], and deletion of MTX2 in human primary fibroblasts leads to reduced MTX1 expression, contributing to mitochondrial dysfunction [47]. Similarly, ALKBH7, an RNA demethylase localized in mitochondria, regulates nascent mitochondrial RNA processing, and its depletion results in mitochondrial impairment [48]. Loss of PARL, a mitochondrial rhomboid protease, leads to aberrant mitochondrial ultrastructure and spermatogenesis failure [49]. A recent study reported that *COX15* deficiency leads to mitochondrial dysfunction and induces ferroptosis in oocytes, ultimately resulting in impaired oocyte development [50]. Our study demonstrated that hnRNPU loss also led to mitochondrial dysfunction. Specifically, deletion of *Hnrnpu* significantly disrupted the transcription of genes associated with mitochondrial function. Notably, the expression levels of *COX15*, *COX1*, *CYTB*, *ND4*, and *Atp4a* were markedly reduced. In addition, the alternative splicing of several mitochondrial‐related genes, including *Mtx1*, *Alkbh7*, and *Parl*, was significantly altered. These findings provide the first evidence that hnRNPU regulates the expression of mitochondrial function‐related genes in oocytes and underscores its importance in mitochondrial function through transcriptional regulation. Importantly, hnRNPU specifically mediates the alternative splicing of mitochondria‐related genes in oocytes, a mechanism that was not thoroughly explored in previous studies. This finding highlights the importance of proper alternative splicing in maintaining oocyte quality and suggests that hnRNPU regulates mitochondrial function through both transcriptional and post‐transcriptional mechanisms. Additionally, the splicing defects observed in key mitochondrial genes further reinforce the critical role of hnRNPU as a modulator of oocyte development through its splicing activity.

Mitochondrial dysfunction is a key determinant of aging, with increasing evidence suggesting a strong link between mitochondrial dysfunction and ovarian aging [51]. A study on the effects of metabolic decline on human ovarian aging has shown that aged oocytes exhibit increased oxidative stress and impaired mitochondrial activity [52]. Another study revealed that the mitochondrial pool and mitochondrial ATP levels are decreased in aged female oocytes, while deletion of *Pdss2* in mouse oocytes results in mitochondrial defects resembling those found in aged females, finally leading to a diminished ovarian reserve [53]. Moreover, evidence suggests that Nicotinamide adenine dinucleotide (NAD^+^) exhibits an age‐dependent decrease in mice. Increasing NAD^+^ levels enhances mitochondrial membrane potential and reduces mitochondrial clustering, eventually attenuating ovarian aging [54]. In our study, we found that the deletion of *Hnrnpu* in growing oocytes results in excessive mitochondria clustering, reduced mitochondrial membrane potential, and elevated ROS levels. These mitochondrial phenotypes closely resemble those observed in aged oocytes, thus highlighting that dysregulation of hnRNPU may accelerate ovarian aging, ultimately leading to infertility. Furthermore, genetic mutations in mitochondria‐related genes, such as those in POLG and MRPS22, have been reported to be relevant to POI. POLG mutation can cause a severe multisystem disorder, and some women had early menopause before the age of 35 [55], whereas MRPS22 mutations directly lead to POI [56]. These findings emphasize the importance of mitochondrial functional integrity in attenuating ovarian aging. While our study demonstrated mitochondrial dysfunction and oocyte maturation arrest in mice with oocyte‐specific *Hnrnpu* deletion, further studies are needed to clarify hnRNPU's potential role in ovarian aging.

Effective communication between oocytes and granulosa cells is crucial for maintaining oocyte quality [57]. Disruption of this bidirectional communication compromises oocyte development and can lead to infertility [58]. Oocytes secrete multiple factors, including GDF‐9, BMP‐15, and FGF‐8, which are essential for the development of both oocytes and granulosa cells [59]. Conversely, granulosa cells regulate oocyte meiotic resumption by providing signaling factors, such as cyclic adenosine monophosphate (cAMP) and cyclic guanosine monophosphate (cGMP) [60]. Since oocytes cannot efficiently metabolize glucose, granulosa cells provide metabolic support by metabolizing glucose for oocyte maturation [61]. This communication relies heavily on intact cell–cell junctions [62]. In our study, oocyte‐specific deletion of *Hnrnpu* in growing oocytes caused widespread transcriptional dysregulation, affecting genes involved in cell adhesion and cell junctional organization. As a result, follicular development was impaired, with few follicles advancing to the antral stage in cKO ovaries. Meanwhile, the expression of N‐cadherin and β‐catenin, two important cell adhesion molecules involved in maintaining the structural integrity of TZPs, was markedly reduced in ZcKO mice (Figure 6A–D). This indicates disrupted oocyte‐granulosa cell interactions. Furthermore, granulosa cell proliferation was significantly decreased, accompanied by an increase in apoptotic cells in ZcKO mice. Our findings suggest the existence of a mechanism by which oocyte‐specific deletion of hnRNPU disrupts the expression of genes essential for oocyte‐granulosa cell communication, impairs granulosa cell proliferation, and promotes their apoptosis, finally contributing to oocyte maturation arrest and female infertility.

hnRNPU, also known as scaffold attachment factor A (SAF‐A), is predominantly localized in the nucleus but is also detected in the cytoplasm [63]. Interestingly, a recent study reported that a fraction of hnRNPU localizes to the outer mitochondrial membrane (OMM) in HCT116 cells, and its depletion impairs the translation of OMM‐localized mRNAs [64]. In our study, oocyte‐specific deletion of hnRNPU led to mitochondrial dysfunction, which was associated with the dysregulation of genes essential for mitochondrial function. While our investigation primarily focused on transcriptional and alternative splicing roles of hnRNPU, it is possible that hnRNPU may also regulate the translation of mitochondrial function‐related genes in oocytes. Additionally, we observed widespread transcriptional and post‐transcriptional dysregulation of mitochondrial genes in ZcKO oocytes; however, the precise mechanisms by which hnRNPU directly modulates mitochondrial activity remain unclear. Although several splicing abnormalities were identified, the specific downstream target transcripts of hnRNPU and their effects on protein expression and mitochondrial function have yet to be fully elucidated. Future studies utilizing proteomic and metabolic profiling as well as RNA‐protein interaction analyses may help to elucidate how hnRNPU‐mediated alternative splicing influences mitochondrial dynamics and oocyte quality, and whether hnRNPU also participates in translational regulation in oocytes. Furthermore, we demonstrated that hnRNPU deletion disrupts oocyte‐granulosa cell communication and induces granulosa cell apoptosis, but the precise molecular mechanisms linking these defects to impaired folliculogenesis and infertility remain to be clarified. Elucidating how loss of hnRNPU in oocytes influences granulosa cell development and function at the molecular level will be an important direction for future research.

In conclusion, our study reveals a novel role for hnRNPU in oocyte maturation by regulating mitochondrial function through transcriptional and alternative splicing mechanisms. hnRNPU also supports the bidirectional communication between oocytes and surrounding granulosa cells. These findings not only deepen our understanding of the molecular mechanisms underlying oocyte development, but also suggest that hnRNPU may represent a promising therapeutic target for female infertility and related diseases, such as POI. Future research should further elucidate the signaling pathways by which hnRNPU regulates mitochondrial activity and oocyte‐granulosa cell communication, thereby advancing our understanding of its potential as a therapeutic target for reproductive health.

## Author Contributions

Jinmei Li, Shenglei Feng, and Juan Dong conceived and designed the study. Jinmei Li, Bei Chen, Yujiao Wen, Yanqing Wu, Kuan Liu, and Jingshou Chen performed all bench experiments. Jinmei Li, Shuangqi Wang, and Shenglei Feng collected and analyzed the data. Yujiao Wen, Bei Chen, and Juan Dong wrote the paper. Juan Dong supervised the project. All authors read and approved the final manuscript.

## Funding

This work was supported by MOST | National Natural Science Foundation of China (NSFC) (82101738, 82401884, and 82401949), HSTD | Natural Science Foundation of Hunan Province (湖南省自然科学基金) (2025JJ50106), Changsha Municipal Natural Science Foundation (kq2502028).

## Ethics Statement

All animal experiments were approved by the Institutional Animal Care and Use Committee (IACUC) of Tongji Medical College, Huazhong University of Science and Technology.

## Conflicts of Interest

The authors declare no conflicts of interest.

## Supporting information


**Figure S1:** Deletion of *Hnrnpu* specifically in oocytes leads to the failure of oocyte maturation. (A) Representative image of oocytes superovulated from 8 weeks old Control and ZcKO mice, respectively. Scale bar = 100 μm. (B) Quantitative analysis of oocytes retrieved from the ampulla of the fallopian tubes in Control, ZcKO and GcKO mice. Data were presented as mean ± SEM. ***p < 0.001. (*n* = 3 biological replicates). (C) Quantitative analysis of oocytes with first polar body extrusion rate in Control and ZcKO mice. Data were presented as mean ± SEM. ***p < 0.001, (*n* = 3 biological replicates) (D) IF staining of α‐tubulin (green) in Control and ZcKO oocytes collected from the ampulla of the fallopian tubes. Nuclei were stained with DAPI. Scale bar = 20 μm. (E) The distribution of F‐actin in MII oocytes from Control and ZcKO. F‐actin was stained with phalloidin (red). Chromosomes were stained with DAPI (blue). Spindles were stained with α‐tubulin (green). Scale bar = 20 μm. Quantitative analysis of abnormal chromosome rate in Control and ZcKO mice was showed in the bottom left. Data were presented as mean ± SEM. ***p < 0.001, (*n* = 3 biological replicates).
**Figure S2:** Severe reduction and abnormal morphology of GV oocytes in Hnrnpu‐GcKO mice. Representative bright‐field images of germinal vesicle (GV) oocytes collected from control and Hnrnpu‐GcKO mice. Control mice yielded abundant GV oocytes with normal morphology, whereas Hnrnpu‐GcKO mice produced very few GV oocytes, which frequently displayed abnormal morphology and poor quality. Scale bars, 100 μm.
**Figure S3:** Analyses of hnRNPU binding to the promoter of its targeted genes. (A) Metagene and heatmap plots of hnRNPU ChIP‐seq signals around transcription start sites (±2 kb). hnRNPU shows strong promoter enrichment, whereas input displays minimal signal. (B) Venn diagram illustrating the overlap between hnRNPU‐bound genes and DEGs. A subset of up‐ and down‐regulated genes is associated with hnRNPU binding. (C) Functional enrichment analysis of DEGs with hnRNPU promoter binding. Dot size reflects gene number, and color reflects log10 (*p* value). (D) Genomic binding of hnRNPU at example genes, visualized using IGV tracks. *Gfpt1* and *Cox15* are down‐regulated in ZcKO, while *Zfp36l2* and *Cdc5l* are up‐regulated.
**Figure S4:** Effect of alternatively spliced events induced by hnRNPU deficiency on the transcripts. (A) Venn diagram showing intersection between alternatively spliced (AS) genes and differentially expressed genes (DEGs) in hnRNPU cKO oocytes. (B) Donut chart showing the predicted effect of exon inclusion on the open reading frame (ORF). Alternatively spliced exons were classified as ORF‐preserving or ORF‐nonpreserving based on whether exon inclusion maintains the coding frame of the transcript. Numbers indicate the total count of exons in each category. (C) Exon ontology analysis of alternatively spliced exons across different protein feature categories. Bars represent the number of exon regions in each category. PTM: post‐translational modification (PTM). Link widths correspond to the number of unique annotated features shared between each gene and category. (D) Two different *Trp53* transcripts caused by alternative splicing corresponding to Figure 4H. (E) IF staining of P53 (red) in adult control and ZcKO ovaries. Nuclei were stained with DAPI. The yellow arrow indicates the nucleus. Scale bar = 100 μm. (F) A Sankey diagram showing the relationships between apoptosis‐related genes (left) and functional feature categories (right).
**Figure S5:** The full Blots and Gels image.

**Table S1:** Primer sequences in this study.

**Table S2:** Antibodies used in this study.

**Table S3:** fsb271445‐sup‐0004‐TableS3.xlsx.

**Table S4:** Total identified hnRNPU‐binding DEGs by analysis of published ChIP‐seq data.

**Table S5:** fsb271445‐sup‐0006‐TableS5.xls.

## Data Availability

All data supporting this study are available within the article and its Supporting Information. Single‐cell RNA‐seq data are deposited in the NCBI SRA (Sequence Read Achieve) database with the accession number PRJNA1377037. Public ChIP‐seq data sets used in this study are accessible through the GEO Series accession number PRJNA376123. Additional datasets or related information are available from the corresponding authors upon reasonable request.
