# Supplementary material for: hnRNPU Safeguards Oocyte Development and Female Fertility via Regulation of Alternative Splicing
Source: FASEB J. 2026 Jan 12;40(2):e71445. doi: 10.1096/fj.202503270R (PMC12794171; doi:10.1096/fj.202503270R)
Supplement: Supplementary file 1 — Figure S1: Deletion of Hnrnpu specifically in oocytes leads to the failure of oocyte maturation. (A) Representative image of oocytes superovulated from 8 weeks old Control and ZcKO mice, respectively. Scale bar = 100 μm. (B) Quantitative analysis of oocytes retrieved from the ampulla of the fallopian tubes in Control, ZcKO and GcKO mice. Data were presented as mean ± SEM. ***p < 0.001. (n = 3 biological replicates). (C) Quantitative analysis of oocytes with first polar body extrusion rate in Control and ZcKO mice. Data were presented as mean ± SEM. ***p < 0.001, (n = 3 biological replicates) (D) IF staining of α‐tubulin (green) in Control and ZcKO oocytes collected from the ampulla of the fallopian tubes. Nuclei were stained with DAPI. Scale bar = 20 μm. (E) The distribution of F‐actin in MII oocytes from Control and ZcKO. F‐actin was stained with phalloidin (red). Chromosomes were stained with DAPI (blue). Spindles were stained with α‐tubulin (green). Scale bar = 20 μm. Quantitative analysis of abnormal chromosome rate in Control and ZcKO mice was showed in the bottom left. Data were presented as mean ± SEM. ***p < 0.001, (n = 3 biological replicates). Figure S2: Severe reduction and abnormal morphology of GV oocytes in Hnrnpu‐GcKO mice. Representative bright‐field images of germinal vesicle (GV) oocytes collected from control and Hnrnpu‐GcKO mice. Control mice yielded abundant GV oocytes with normal morphology, whereas Hnrnpu‐GcKO mice produced very few GV oocytes, which frequently displayed abnormal morphology and poor quality. Scale bars, 100 μm. Figure S3: Analyses of hnRNPU binding to the promoter of its targeted genes. (A) Metagene and heatmap plots of hnRNPU ChIP‐seq signals around transcription start sites (±2 kb). hnRNPU shows strong promoter enrichment, whereas input displays minimal signal. (B) Venn diagram illustrating the overlap between hnRNPU‐bound genes and DEGs. A subset of up‐ and down‐regulated genes is associated with hnRNPU binding. (C) Functio [file FSB2-40-e71445-s006.docx]

**Supplementary Figures**

**Supplementary Figure 1**

**
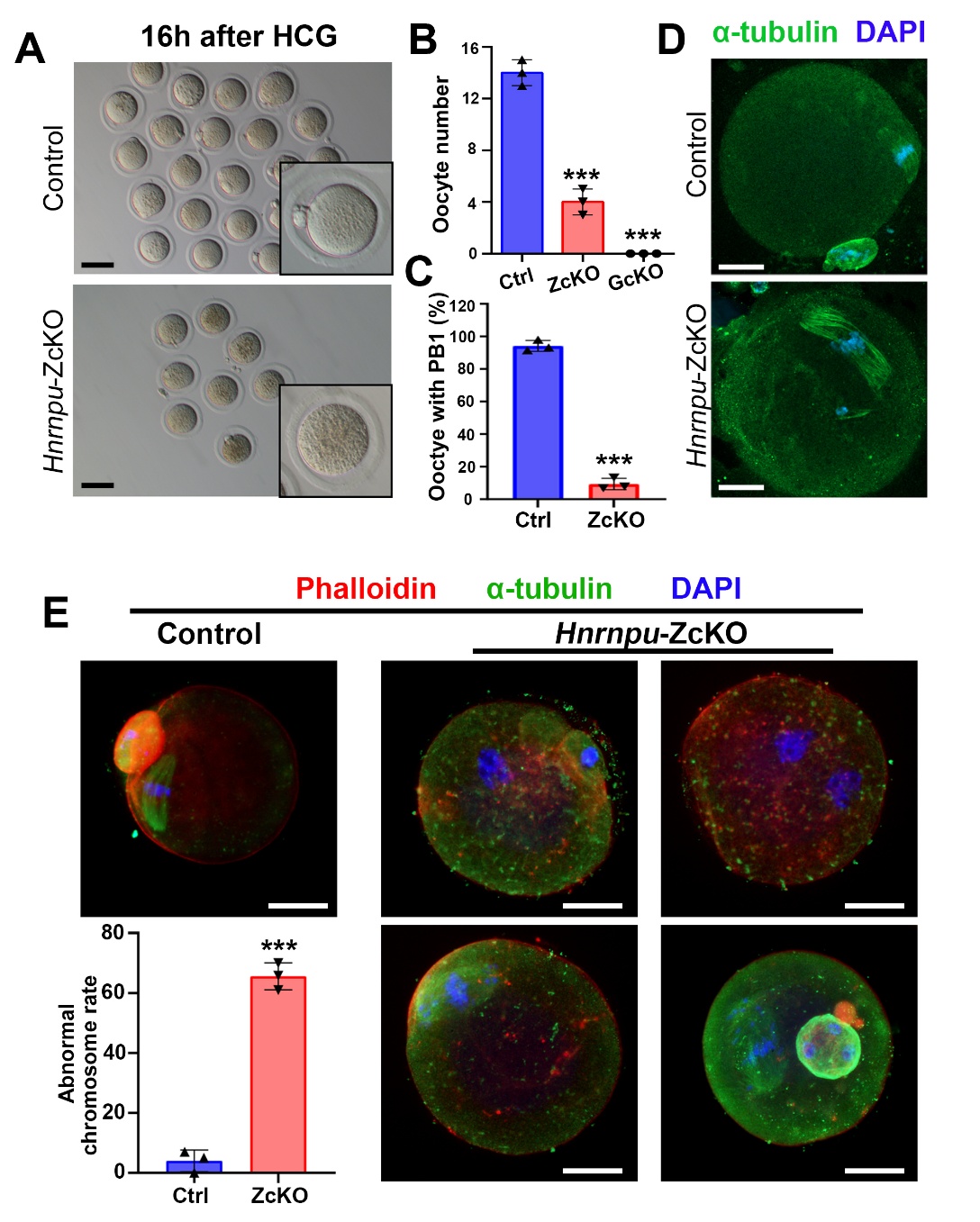
**

**Supplementary Figure 1.** Deletion of *Hnrnpu* specifically in oocytes leads to the failure of oocyte maturation. (**A**) Representative image of oocytes superovulated from 8 weeks old Control and ZcKO mice, respectively. Scale bar = 100 µm. (**B**) Quantitative analysis of oocytes retrieved from the ampulla of the fallopian tubes in Control, ZcKO and GcKO mice. Data were presented as mean ± SEM. *** *p* < 0.001. (n = 3 biological replicates). (**C**) Quantitative analysis of oocytes with first polar body extrusion rate in Control and ZcKO mice. Data were presented as mean ± SEM. *** *p* < 0.001. (n=3 biological replicates) (**D**) IF staining of α-tubulin (green) in Control and ZcKO oocytes collected from the ampulla of the fallopian tubes. Nuclei were stained with DAPI. Scale bar = 20 µm. (**E**) The distribution of F-actin in MII oocytes from Control and ZcKO. F-actin was stained with phalloidin (red). Chromosomes were stained with DAPI (blue). Spindles were stained with α-tubulin (green). Scale bar = 20 μ m. Quantitative analysis of abnormal chromosome rate in Control and ZcKO mice was showed in the bottom left. Data were presented as mean ± SEM. *** *p* < 0.001. (n=3 biological replicates).

**Supplementary Figure 2**


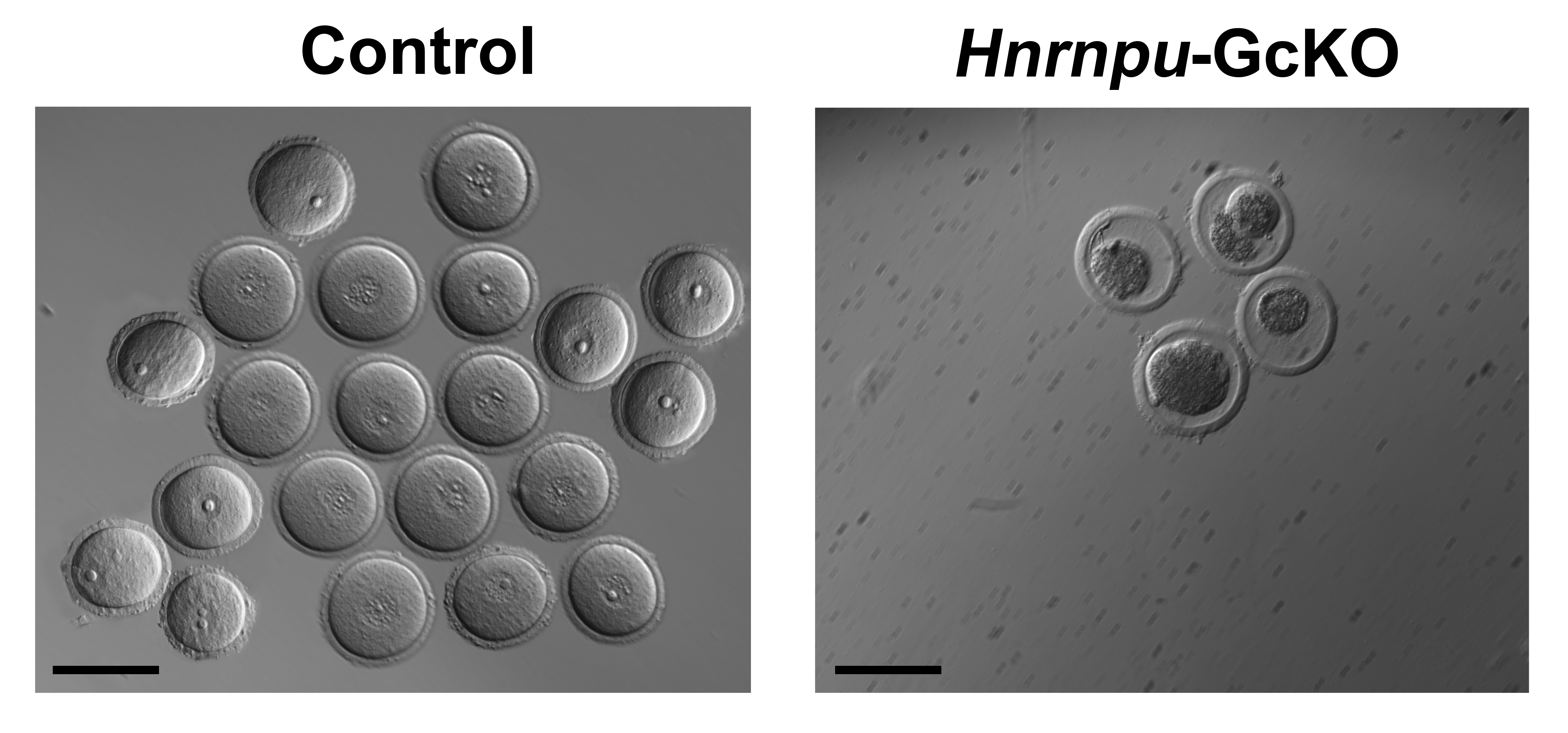


**Supplementary Figure 2.** Severe reduction and abnormal morphology of GV oocytes in *Hnrnpu*-GcKO mice. Representative bright-field images of germinal vesicle (GV) oocytes collected from control and *Hnrnpu*-GcKO mice. Control mice yielded abundant GV oocytes with normal morphology, whereas *Hnrnpu*-GcKO mice produced very few GV oocytes, which frequently displayed abnormal morphology and poor quality. Scale bars, 100 µm.

**Supplementary Figure 3**


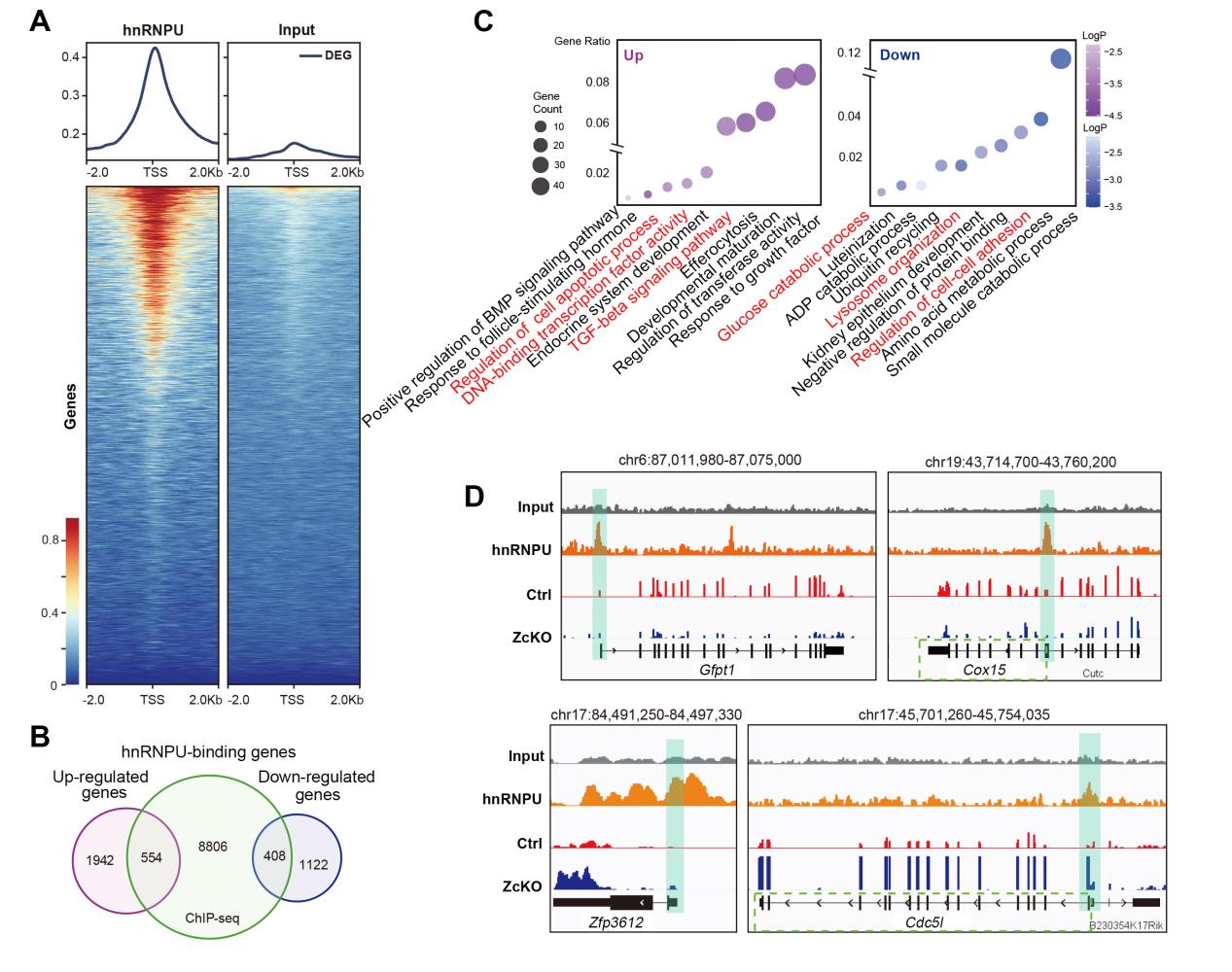
**Supplementary Figure 3.** Analyses of hnRNPU binding to the promoter of its targeted genes. **(A)** Metagene and heatmap plots of hnRNPU ChIP-seq signals around transcription start sites (±2 kb). hnRNPU shows strong promoter enrichment, whereas input displays minimal signal. **(B)** Venn diagram illustrating the overlap between hnRNPU-bound genes and DEGs. A subset of up- and down-regulated genes is associated with hnRNPU binding. **(C)** Functional enrichment analysis of DEGs with hnRNPU promoter binding. Dot size reflects gene number, and color reflects log10 (P value). **(D)** Genomic binding of hnRNPU at example genes, visualized using IGV tracks.. *Gfpt1* and *Cox15* are down-regulated in ZcKO, while *Zfp36l2* and *Cdc5l* are up-regulated.

**Supplementary Figure 4**


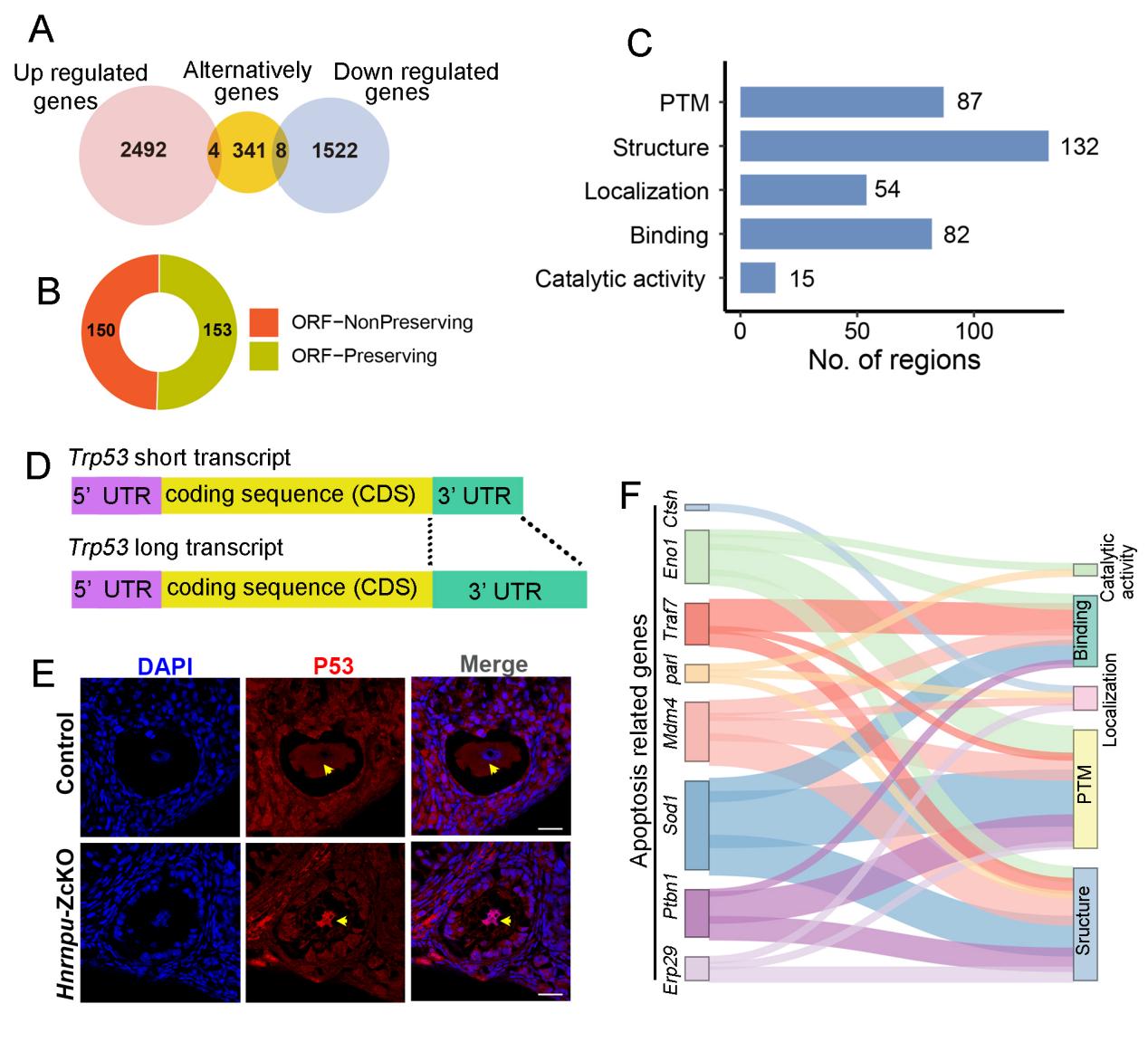
**Supplementary Figure 4.** Effect of alternatively spliced events induced by hnRNPU deficiency on the transcripts. **(A)** Venn diagram showing intersection between alternatively spliced (AS) genes and differentially expressed genes (DEGs) in hnRNPU cKO oocytes. **(B)** Donut chart showing the predicted effect of exon inclusion on the open reading frame (ORF). Alternatively spliced exons were classified as ORF-preserving or ORF-nonpreserving based on whether exon inclusion maintains the coding frame of the transcript. Numbers indicate the total count of exons in each category. **(C)** Exon ontology analysis of alternatively spliced exons across different protein feature categories. Bars represent the number of exon regions in each category. PTM: post-translational modification (PTM). Link widths correspond to the number of unique annotated features shared between each gene and category. **(D)** Two different *Trp53* transcripts caused by alternative splicing corresponding to Figure 4H. **(E)** IF staining of P53 (red) in adult control and ZcKO ovaries. Nuclei were stained with DAPI. The yellow arrow indicates the nucleus. Scale bar = 100 µm. **(F)** A Sankey diagram showing the relationships between apoptosis-related genes (left) and functional feature categories (right).

**Supplementary Figure 5: The full Blots and Gels image**

**
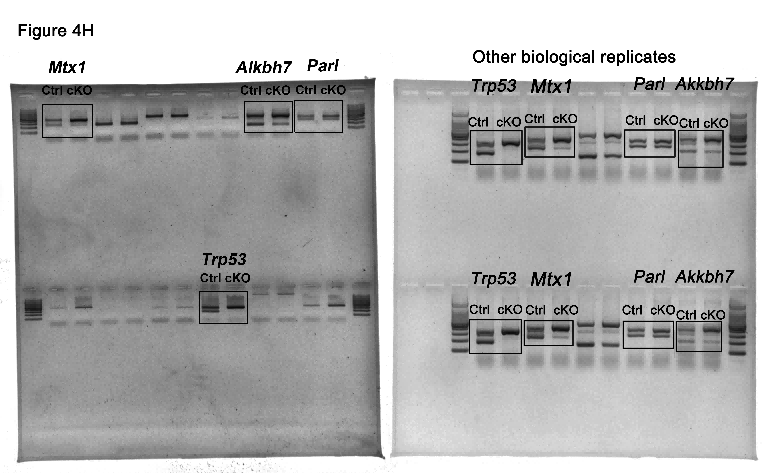
**
