# Supplementary material for: hnRNPU Safeguards Oocyte Development and Female Fertility via Regulation of Alternative Splicing
Source: FASEB J. 2026 Jan 12;40(2):e71445. doi: 10.1096/fj.202503270R (PMC12794171; doi:10.1096/fj.202503270R)
Supplement: Supplementary file 2 — Table S1: Primer sequences in this study. [file FSB2-40-e71445-s004.docx]

**Supplementary Table 1. Primer sequences in this study**

| **Gene name** | **Sequence (5’-3’)** | **Application** |
| --- | --- | --- |
| Gdf9-cre-F | CAGGTTTTGGTGCACAGTCA | Genotyping |
| Gdf9-cre-R | GGCATGCTTGAGGTCTGATTAC |  |
| Hnrnpu-loxP-F | TCAGCAGCGAATGGTATCAA |  |
| Hnrnpu-loxP-R | GAAAGCAGCCATGTTTTACACTT |  |
| Zp3-cre-F | GCCTGCATTACCGGTCGATGC |  |
| Zp3-cre-R | CAGGGTGTTATAAGCAATCCC |  |
| Atp4a-qPCR-F | TGCTGACTACTTCACGGCCAT | qRT-PCR |
| Atp4a-qPCR-R | ACATCTGCGATCTGGCACA |  |
| Cox15-qPCR-F | TGCTGACTACTTCACGGCCAT |  |
| Cox15-qPCR-R | ACATCTGCGATCTGGCACA |  |
| COX1-qPCR-F | ACCCAATTCTCTACCAGCATC |  |
| COX1-qPCR-R | AGTAAGCTCGTGTGTCTACATC |  |
| CYTB-qPCR-F | CTCCTCTTCCTCCACGAAA |  |
| CYTB-qPCR-R | ATAGGAAATATCATTCGGGTT |  |
| Edn1-qPCR-F | ATCAAACGGCCCATGAACGCC |  |
| Edn1-qPCR-R | GGATAATCAGCCATGTGCTTGAG |  |
| Epha3-qPCR-F | TGTAAGGAAATCTTCACAGGC |  |
| Epha3-qPCR-R | CATCTTCCCGTTTCTCACA |  |
| Epha5-qPCR-F | AGCAATCAAAATGGGTCGGTA |  |
| Epha5-qPCR-R | TCATCTCTTGAAGGCTGCTC |  |
| Fshr-qPCR-F | CAAAGGTCTATTCCCTGCCCAA |  |
| Fshr-qPCR-R | GTCATATCATCAATATCTTGCCT |  |
| Gck-qPCR-F | TGCAACGCCTGCTACATGGA |  |
| Gck-qPCR-R | CTCTACCAGCTTGAGCAGCAC |  |
| Gfpt1-qPCR-F | AGACGCAAAGAGATCATGCT |  |
| Gfpt1-qPCR-R | CCCGCCCCATTATCAGGAC |  |
| Gpi1-qPCR-F | CCTTGCTGCCCTATGACCAGT |  |
| Gpi1-qPCR-R | ATGAGCTGGTAGAATGCATGTTG |  |
| Ncam1-qPCR-F | CACAGAGCCCAACGAGACCA |  |
| Ncam1-qPCR-R | ATTCTCTTTTGTTTGTGTGGCAT |  |
| ND4-qPCR-F | CGCTTCATGATCTAACAAC |  |
| ND4-qPCR-R | AGATGCGAATTATTCCGTA |  |
| Notch1-qPCR-F | TTGCCAGACCAACATCAACGA |  |
| Notch1-qPCR-R | TTCAGACTCCTTGCATACCCC |  |
| Npr2-qPCR-F | CCTTTATACCTGCTTTGATGCC |  |
| Npr2-qPCR-R | CCATTCGAGCAATTTCTGGTG |  |
| Tgfbr3-qPCR-F | AGTCCAGTCCGGTTCCTCC |  |
| Tgfbr3-qPCR-R | GCTCTGAGTGCTCCCTATGCT |  |
| Alkbh7-F | CCGTGGTGCATCCAGGCTTC | RT-PCR |
| Alkbh7-R | CTTCCTGGCCCCATCCCCTC |  |
| MTX1-F | GGCCGTGCTGACCTATACCA |  |
| MTX1-R | CCTCGTTCTCTGATTTGTGCT |  |
| Parl-F | TTGATGGCATAAAAGCTGACT |  |
| Parl-R | AAGTATCTGATCATGGTTCGC |  |
| Trp53-F | GATGCCCATGCTACAGAGGA |  |
| Trp53-R | GGATAAATGCAGACAGGCTT |  |
