# Supplementary material for: hnRNPU Safeguards Oocyte Development and Female Fertility via Regulation of Alternative Splicing
Source: FASEB J. 2026 Jan 12;40(2):e71445. doi: 10.1096/fj.202503270R (PMC12794171; doi:10.1096/fj.202503270R)
Supplement: Supplementary file 3 — Table S2: Antibodies used in this study. [file FSB2-40-e71445-s005.docx]

**Supplementary Table 2. Antibodies used in this study**

| **Antibodies** | **Species** | **Concentration** | **Company** | **Cat No.** |
| --- | --- | --- | --- | --- |
| DDX4 | Rabbit | 1:600 | Abcam | ab13840 |
| α-Tubulin | Mouse | 1:100 | Proteintech | 14555-1-AP |
| KI67 | Rabbit | 1:400 | Abcam | ab15580 |
| hnRNPU | Rabbit | 1:200 | Abclonal | A3917 |
| hnRNPU | Mouse | 1:100 | Santa Cruz | sc-32315 |
| β-catenin | Rabbit | 1:200 | Thermo Fisher | 22-7263 |
| N-cadherin | Mouse | 1:100 | Cell signaling technology | 14215 |
| P53 | Rabbit | 1:200 | Proteintech | 10442-1-AP |
| IFKine™Green Donkey Anti-Mouse IgG | Donkey | 1:500 | Abbkine | A24211 |
| IFKine™ Red Donkey Anti-Mouse IgG | Donkey | 1:500 | Abbkine | A24411 |
| IFKine™Green Donkey Anti-Rabbit IgG | Donkey | 1:500 | Proteintech | A24221 |
| IFKine™ Red Donkey Anti-Rabbit IgG | Donkey | 1:500 | Abbkine | A24421 |
